# A nonhuman primate model for genital herpes simplex virus 2 infection that results in vaginal vesicular lesions, virus shedding, and seroconversion

**DOI:** 10.1371/journal.ppat.1012477

**Published:** 2024-09-03

**Authors:** Kening Wang, Tristan Jordan, Kennichi Dowdell, Richard Herbert, Ian N. Moore, David M. Koelle, Jeffrey I. Cohen

**Affiliations:** 1 Medical Virology Section, Laboratory of Infectious Diseases, National Institute of Allergy and Infectious Diseases, National Institutes of Health, Bethesda, Maryland, United States of America; 2 Experimental Primate Virology Section and; 3 Infectious Disease Pathogenesis Section, Comparative Medicine Branch National Institute of Allergy and Infectious Diseases, National Institutes of Health, Bethesda, Maryland, United States of America; 4 Department of Medicine, University of Washington, Seattle, Washington, United States of America; 5 Department of Laboratory Medicine and Pathology, School of Medicine, University of Washington, Seattle, Washington, United States of America; 6 Department of Global Health, School of Medicine, University of Washington, Seattle, Washington, United States of America; 7 Vaccine and Infectious Disease Division, Fred Hutchinson Cancer Research Center, Seattle, Washington, United States of America; 8 Benaroya Research Institute, Seattle, Washington, United States of America; University of Wisconsin-Madison, UNITED STATES OF AMERICA

## Abstract

The most commonly used animal models for evaluating the efficacy of HSV-2 candidate vaccines are mice and guinea pigs. While numerous HSV-2 vaccine candidates have been tested in these animals and were effective in reducing disease and mortality, these results did not predict the effectiveness of the vaccines in human trials. Infection of rhesus macaques rarely results in lesions or HSV-2 specific antibody responses. In seeking an animal model that better recapitulates human disease and that might be more predictive of the efficacy of prophylactic vaccines than mice and guinea pigs, we evaluated Cebus apella (C. apella), a New World primate, in an HSV-2 genital infection model. Infectious HSV-2 was cultured from vaginal swabs from all 4 animals for 9–14 days after intravaginal inoculation of HSV-2 seronegative monkeys. Two of 4 monkeys had vesicular lesions in the vagina or vulva. No neurological symptoms were noted. Recurrent lesions and HSV-2 DNA shedding after acute disease resolved was infrequent. UV irradiation of the genital area did not induce recurrent genital lesions or virus shedding. All 4 monkeys developed HSV-2 neutralizing antibodies as well as virus-specific CD4 and CD8 T cell responses. Reinfection of animals 15 to 19 months after primary infection did not result in lesions; animals had reduced virus shedding and a shorter duration of shedding compared with that during primary infection, suggesting that primary infection induced protective immunity. Primary fibroblasts from C. apella monkeys supported the growth of HSV-2 in vitro; in contrast, HSV-2 did not replicate above the titer of the input inoculum in fibroblasts from rhesus macaques. These observations suggest that the C. apella monkey has potential to serve as a model for evaluating the efficacy of prophylactic vaccines, antivirals, or monoclonal antibodies to HSV-2.

## Author summary

Herpes simplex virus 2 (HSV-2) is a cause of genital herpes, neonatal herpes, and herpes encephalitis. The virus persists in the nervous system for life and periodically reactivates resulting in recurrent genital lesions and can be transmitted to susceptible individuals. Currently there is no cure for the disease and no licensed vaccine to prevent infection. Numerous HSV-2 vaccine candidates have been tested in animal models, mostly in mice and guinea pigs, and vaccine efficacy observed in these animals has been poorly predictive for effectiveness of HSV-2 vaccines in humans. Thus, there is a need for an animal model that better recapitulates human disease and is more predictive for the efficacy of prophylactic vaccines. We evaluated an HSV-2 genital infection model using Cebus apella, a New World primate. Infectious HSV-2 was cultured from vaginal swabs from all 4 animals for 9–14 days after intravaginal inoculation. The animals developed symptoms similar to most humans with genital vesicular lesions and no neurological signs. They also developed antibody and T cell responses to HSV-2. Prior infection generally reduced reinfection. These observations suggest that Cebus apella monkeys have the potential to serve as a good model for evaluating the efficacy of prophylactic HSV-2 vaccines.

## Introduction

Herpes simplex virus 2 (HSV-2) causes multiple diseases including genital herpes, neonatal herpes which can involve the skin, brain, and other organs, herpes encephalitis, and severe local or disseminated disease in immunocompromised individuals. Genital herpes infection increases HIV acquisition and transmission [1,2]. In the past three decades, multiple HSV-2 candidate vaccines have been tested in clinical trials; however, none have shown consistent efficacy in humans or advanced to licensure [3].

The most commonly used animal models for evaluating HSV-2 vaccines are mice and guinea pigs. Various HSV-2 vaccine candidates, including glycoprotein D (gD) and gB subunit vaccines [4], replication-defective HSV-2 [5,6], single-cycle HSV-2 [7,8], attenuated replication-competent HSV [9,10], and mRNA encoding HSV-2 glycoprotein vaccines [11] have been tested in these animals and were efficacious to prevent disease. Of these vaccine candidates, gD or gD/gB subunit vaccines have been most extensively tested for their efficacy as prophylactic vaccines in human trials [12–14]. Despite their ability to provide nearly complete protection of mice and guinea pigs from disease, they were not sufficiently efficacious to proceed to licensure.

Mice infected intravaginally with HSV-2 develop local inflammation, shed virus, and develop latent infections, but do not have spontaneous recurrences. In contrast, guinea pigs develop vesicular genital lesions similar to those seen in humans and the disease frequently recurs with lesions [15]. Mice and guinea pigs frequently develop neurological symptoms including hindlimb paralysis and urinary and fecal retention which can be fatal [15,16]; these complications are extremely rare in humans. Thus, protecting mice or guinea pigs from these complications is not an ideal endpoint as a model for human infection. Instead, prevention of genital lesions and virus shedding, which are common manifestations of genital HSV-2 in humans, are more appropriate endpoints in animal studies.

The immune systems of mice and guinea pigs are different from humans. For example, in C57BL/6 mice, the cytotoxic T cell response to HSV infection is dominated by amino acid residues 498 to 505 of gB (gB498-505) [17], while this is not seen in humans. Other peptide epitopes recognized by T cells also differ among species and within human populations, due to MHC variability. In addition, HSV proteins important for evading immune responses in humans may not inhibit immune responses in mice or guinea pigs. For example, the HSV immune evasion protein ICP47 blocks human, but not mouse, transporter associated with antigen presentation (TAP) protein [18]. In addition, HSV glycoprotein E which acts as an immunoglobulin G (IgG) Fc receptor binds to the Fc domain of human IgG, but not mouse IgG [19].

HSV-2 is thought to have been transmitted from an ancestor of chimpanzees to an extinct precursor of modern humans about 1.6 million years ago [20]. Thus, HSV-2 has coevolved with non-human primates and thus these animals may serve as better models than mice or guinea pigs to predict the efficacy of vaccines in humans. HSV-2 vaginal infection of non-human primates was investigated in 1970s when HSV-2 infection was thought to be a cause of cervical cancer [21]. While squirrel monkeys (Saimiri sciureus) could not be infected with HSV-2 [22,23], baboons (Papio cynocephalus) could be infected, but did not develop vesicular lesions [24]. Initial studies with rhesus monkeys indicated that they could not be infected [22,23], but a more recent study showed that a minority of the animals shed virus for only a few days after infection and did not develop genital lesions [25]. On the other hand, owl monkeys (Aotus trivirgatus) could readily be infected with HSV-1 via conjunctiva or cornea inoculation, but the animals died of overwhelming infection and with necrosis of the liver and adrenal glands soon after inoculation [26].

In contrast to other nonhuman primates, Cebus monkeys (C. albifrons or C. apella) were found to be susceptible to HSV-2 infection and developed vesicular genital and skin lesions without neurologic complications [22,23,27,28]. Some the animals could be reinfected, despite development of neutralizing antibody. HSV-2 could be isolated by cocultivation from two sacral dorsal root ganglia in vitro from one animal, but not from autonomic ganglia. About 20% of male Cebus became infected after being housed with infected Cebus female monkeys. When these studies were done in the 1970’s HSV was thought to be associated with cervical cancer and hundreds of animals were infected to look for development of cervical carcinoma. While some of the animals developed cellular atypia and dysplasia, none developed cancer and human papillomavirus, not HSV, was been shown to be the etiologic agent of cervical cancer. Thus, the Cebus monkey model of HSV was not pursued further at the time. More recently, the very limited use of Cebus monkeys in medical research and the diminished supply of these animals in the last several years has made procuring these animals difficult.

As noted above, while several HSV vaccines have been shown to be highly efficacious in mice and guinea pigs, these vaccines have failed in human trials. Thus, we reevaluated C. apella monkeys with improved diagnostic and immunologic techniques, for their susceptibility to HSV-2 and their clinical, immunologic, and virologic response to vaginal infection to determine if they might be an alternative model to mice and guinea pigs for testing vaccines or monoclonal antibodies.

## Results

### C. apella monkeys acutely infected with HSV-2 infection develop vesicular genital lesions and shed virus for up to 2 weeks

Four female C. apella monkeys, ranging from 1.0 to 2.6-years old and whose serum was negative for HSV-2 neutralizing antibody, were infected intravaginally with HSV-2. Two animals (A-333 and J-333) were infected with a plaque purified isolate from HSV-2 333, which has been passaged numerous times in Vero cells. Two monkeys (F- and K-Bethesda) were infected with HSV-2 Bethesda, a clinical isolate which had been only passaged four times in human fibroblasts and once in Vero cells and was not plaque purified. Animals received medroxyprogesterone acetate (Depo-provera) 5 days before inoculation to synchronize the menstrual cycles of the animals, so that more consistent responses could be obtained from the limited number of animals available. Physical examinations, blood draws, and vaginal swabs were obtained at various times points before and after virus inoculation (**Table 1).** Due to reduction of food consumption and weight loss in 2 monkeys after 5 consecutive days of sedation during procedures, physical examinations and vaginal swabs were reduced to 3 times a week for the second week after infection and then twice weekly thereafter.

**Table 1:** Outline of animal primary infection and monitoring procedures.

Day	-5	0	1	2	3	4	7	9	11	14	Twice a week to Day 109
Depo-provera	X										
Intravaginal Infection (pfu)		2x10^6^									
Blood draw for serum (ml)	1									1	
Blood draw for plasma (ml) and PBMC		0.5	0.5	0.5	0.5	0.5	0.5	0.5	0.5	0.5	
Vaginal and other lesion swabs for virus culture	X	X	X	X	X	X	X	X	X	X	X
Vaginal and other lesion swabs for PCR	X	X	X	X	X	X	X	X	X	X	X
Exam: Overall health; genital and other lesions	X	X	X	X	X	X	X	X	X	X	X

**Fig 1:**
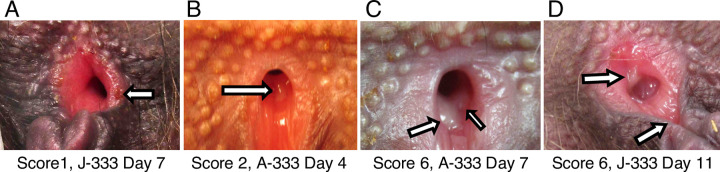
Lesions observed during acute infection with scoring system for HSV-2 infected C. apella monkeys. Vesicular lesions were seen predominantly inside the vagina, with a few on the clitoris. Individual small, medium, or large vesicular lesions were scored as 1, 2, or 3, respectively and if more than one lesion was observed, the sum of the individual scores was used. Arrows point to the lesions. (A) A small lesion at the edge of the vagina orifice of monkey J-333 on day 7, scored as 1. (B) A medium lesion inside the vagina of monkey A-333 on day 4, scored as 2. (C) Two large lesions inside the vagina of monkey A-333 on day 7, scored as 6 (3 + 3). (D) One large lesion inside the vagina and one large lesion on the clitoris of monkey J-333 on day 11, scored as 6 (3 + 3).

**Fig 2:**
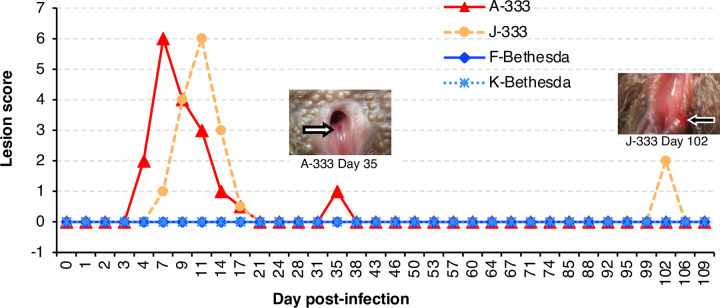
Lesion scores over time in C. apella monkeys after primary infection. The genital area of monkeys was examined on indicated days in the figure after infection. Scoring system is described in Fig 1. Lesions were observed at days 35 and 102 during the latent phase of infection; arrows point to small lesions. Data derived from **S1 Data**.

The two monkeys infected with HSV-2 333 developed vesicular lesions in the vagina **(Fig 1)** and vulva. Individual lesions were scored on a scale from 1 to 3 with the total score on each day defined as the total of the number lesions multiplied by the score of each lesion. No vesicular lesions were observed in animals infected with HSV-2 Bethesda, although they shed virus (see below). Vesicular lesions began in the vagina of monkey A-333 on day 4 after infection, peaked on day 7 with a score of 6, and were all resolved by day 21 (**Fig 2**). Vesicular lesions were first observed in the vagina of monkey J-333 on day 7, peaked on day 11 with a score of 6, and all were healed by day 21 (**Fig 2**). Lesions persisted for a mean of 14 days. These two monkeys had mild weight loss (5–7%) at the time lesions were present compared to their weight on day 0. None of the animals had skin lesions, oral lesions, diarrhea or constipation, or behavioral changes. None developed neurological complications such as hind-limb paralysis, in contrast to what has been observed in mice [29] and guinea pigs [15] after intravaginal infection.

**Fig 3:**
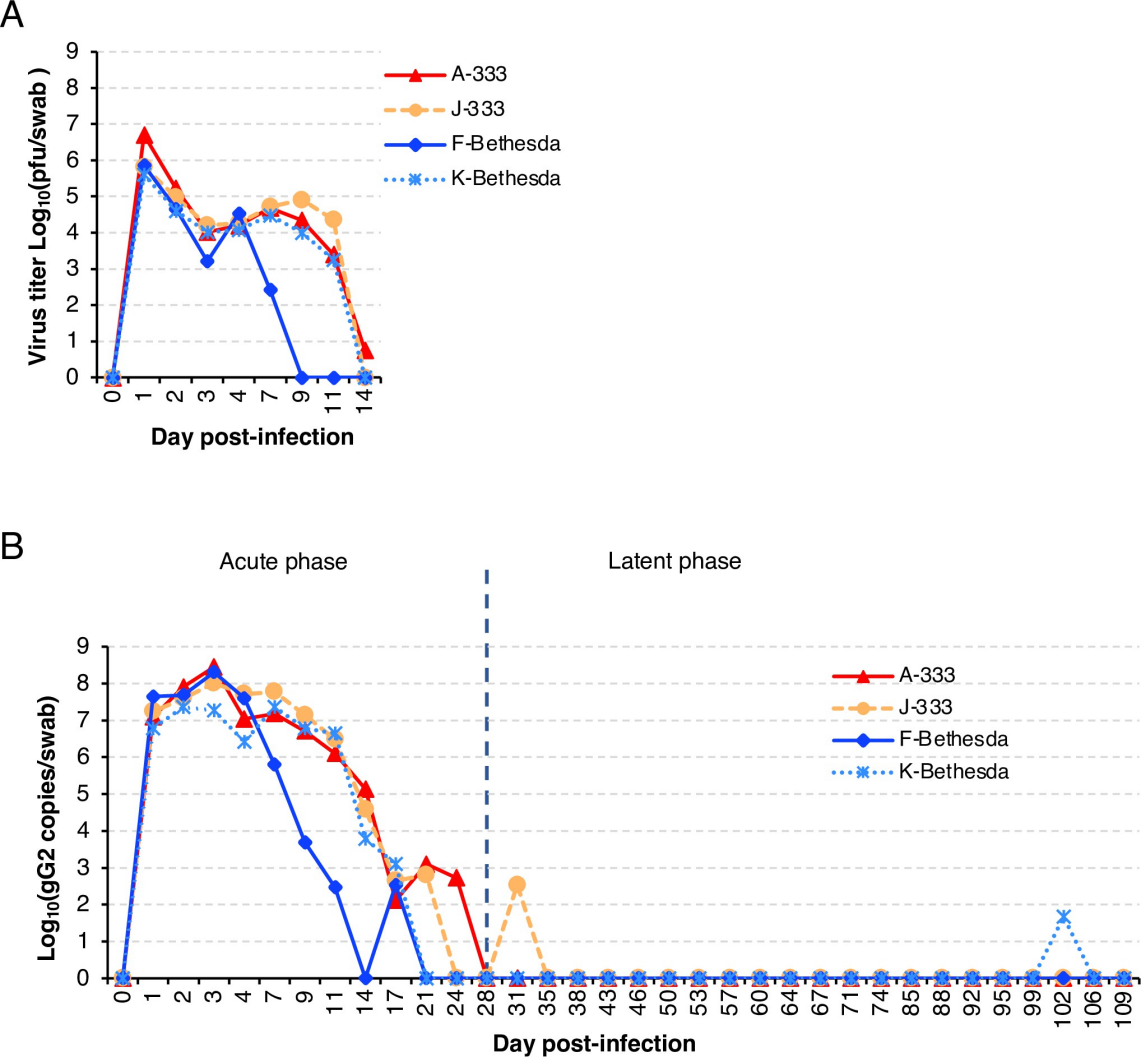
C. apella monkeys shed infectious HSV-2 after intravaginal infection and developed a few lesions during the latent phase of infection. Two vaginal swabs were taken on each day post-infection as indicated in the figures. (A) Titer of HSV-2 from vaginal swabs based on plaque counts on Vero cells. (B) Copy number of HSV-2 DNA from vaginal swab based on real time PCR with primers and probe specific for the HSV-2 gG gene. Day 0 to day 27 is considered the acute phase of infection and day 28 (vertical dotted line) and later is the latent phase. Data derived from **S2 Data**.

Vaginal swabs from all 4 monkeys were HSV-2 culture positive beginning on day 1 after infection, and titers ranged from 4.1 x 10^5^ to 5.1 x 10^6^ pfu/swab (**Fig 3A**). The virus titer fell rapidly on days 2 and 3, to about 1.0 x 10^4^ to 1.6 x 10^4^ pfu/swab on day 3, with the exception of monkey F-Bethesda whose titer declined to 1.6 x 10^3^ pfu/swab. In 3 monkeys (A-333, J-333, K-Bethesda) the vaginal swab titers of HSV-2 were maintained at a similar level from days 3 to 9, and then gradually decreased and were undetectable by day 14 in 2 monkeys, while one monkey (A-333) still had a titer of 6 pfu/swab. The dynamics of the vaginal swab virus titers of one of the animals infected with HSV-2 Bethesda (F-Bethesda) was different and the virus titer decreased after day 4 and was undetectable by day 9. We subsequently found that HSV-2 Bethesda produced smaller plaques in Vero cells than HSV-2 333 suggesting that HSV-2 Bethesda is impaired for cell-to-cell spread which may explain why monkeys infected with HSV-2 Bethesda did not develop visible lesions, but still shed virus.

HSV-2 DNA shedding was also tested by real-time qPCR of DNA from vaginal swabs. As expected, viral DNA shedding persisted longer than shedding of infectious virus (**Fig 3B**). Monkey F-Bethesda had the earliest clearing of both infectious virus (on day 9) and viral DNA (on day 21). By day 14, no infectious virus could be detected from vaginal swabs from three animals, but viral DNA was still detected at low levels for 7 to 10 additional days. While the titer of infectious virus in vaginal swabs peaked on day 1 and gradually fell on day 2 and day 3, the HSV-2 DNA copy number increased from day 1 to day 3 and then gradually fell; by day 28 vaginal swabs from all 4 animals were HSV-2 DNA negative. The longer time to the peak of HSV-2 DNA compared to infectious virus may be due the longer time to clear viral DNA compared to infectious virus. No infectious virus could be detected from swabs when HSV-2 DNA copies were ≤ 37,800/swab, while infectious virus was detected from all swabs when HSV-2 DNA copies were ≥138,000/swab. Thus, the minimum number of HSV-2 DNA copies/swab corresponding to detectable infectious virus was between about 3.78x10^4^ to 1.38x10^5^ copies/swab. The first day vaginal swabs from all 4 monkeys were HSV-2 DNA negative was day 28 post-infection; therefore, we defined the last day of acute infection as day 27 with latent infection beginning the next day.

### HSV-2 DNA is present in the plasma of C. apella monkeys infected intravaginally with HSV-2

**Fig 4:**
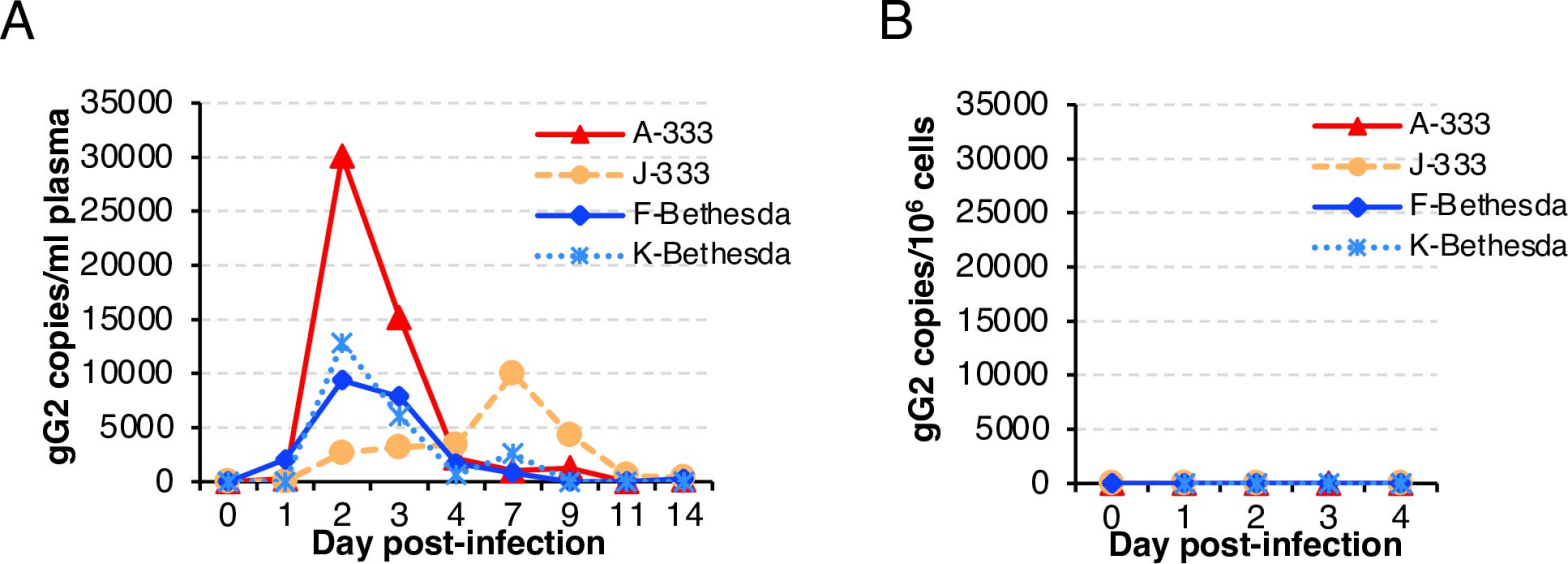
HSV-2 DNA is detected in the plasma, but not in PBMCs of C. apella monkeys after intravaginal infection. 0.5 ml blood samples were collected daily after infection and PBMCs were separated from plasma by centrifugation of whole blood in Ficoll. DNA was isolated from PBMCs and 200 ul of plasma was assayed by real time PCR with primers and probe specific for the HSV-2 gG gene. (A) HSV-2 DNA copies per ml of plasma. (B) HSV-2 DNA copies per 10^6^ PBMCs. Data derived from **S3 Data**.

To determine if HSV-2 DNA is present in blood after intravaginal infection of C. apella monkeys, blood samples were collected immediately before virus inoculation (day 0) and on days 1 to 4 then 7, 9, 11, and 14 days after infection. HSV-2 DNA was detected in the plasma of all four monkeys. Three animals had peak levels of viral DNA on day 2 with 3.0 x 10^4^, 9.4 x 10^3^, and 1.3 x 10^4^ copies of HSV-2 genome/ml of plasma (monkeys A-333, F-Bethesda, K-Bethesda, respectively) (**Fig 4A**), and the peak level of viral DNA was 2 days before the first vaginal lesion was detected. Beginning at day 4, these animals were persistently positive at a much lower level for HSV-2 DNA in the plasma for an additional 4 to 5 days. In contrast, HSV-2 DNA in the plasma of one monkey that received HSV-2 333 (animal J-333) was detectable on day 2, and peaked on day 7 at 1 x 10^4^ copies of HSV-2 genome/ml of plasma, the same day the monkey developed a vaginal lesion. We tested the remaining available plasma, obtained on days 7, 9, 11,14 after infection, for infectious virus by culturing 100 ul of plasma on Vero cells. All of these cultures were negative for infectious HSV-2. It is possible that plasma obtained on days 2 or 3, which had much higher levels of HSV-2 DNA than later time points, may have had infectious virus. Unlike plasma, DNA extracted from peripheral blood mononuclear cells (PBMCs) collected on days 1 to 4 was HSV-2 DNA negative (**Fig 4B**).

### Spontaneous reactivation of HSV-2 is rarely detected in C. apella monkeys

We continued to monitor the infected monkeys after day 27 (considered the end of the acute phase of infection since all 4 monkeys were PCR negative at day 28) twice weekly for lesions and obtained vaginal swabs for qPCR of HSV-2 DNA up to day 109 (**Fig 3B**). Thereafter, 2 out of 84 swabs were PCR-positive for viral DNA; monkey J-333 had 228 copies/swab on day 31 and monkey K-Bethesda had 32 copies/swab on day 102. A small non-vesicular lesion was observed in the vagina of monkey A-333 on day 35 and in monkey J-333 on day 102 (**Fig 2**); however, swabs taken from these lesions were PCR-negative for HSV-2 DNA. These lesions may have been unrelated to HSV-2 or the swabs may have been obtained late in the course of reactivation resulting in a negative PCR for HSV-2 DNA. These observations indicate that spontaneous reactivation of HSV-2 in the C. apella monkey genital model, in which animals received a single dose of virus, is infrequent.

### Ultraviolet (UV) light irradiation does not induce reactivation of HSV-2 in C. apella monkeys

Due to the very low rate of spontaneous reactivation in the monkeys, we tried to induce HSV-2 reactivation by UV irradiation of the perineum of the animals. Prior studies of humans have shown that UV irradiation at the site of previous vesicular HSV-2 lesions can induce virus reactivation [30, 31]. Animals were subjected to UV irradiation at a dose of 600 mJ/cm^2^/min for 2 minutes on day 214 after initial infection. Vaginal swabs were obtained immediately before irradiation, and on days 3, 4, 5, 7 after irradiation and cultured for infectious virus in Vero cells. Day 3 was the earliest day chosen for swab collection based on a prior study in humans [30]. The day after irradiation, erythema was observed in the perineum of the monkeys indicating that UV exposure had been sufficient to induce skin irritation; however, all swabs were negative for HSV-2 by culture and by PCR for viral DNA. UV irradiation was repeated on day 235 with the duration of exposure increased to 4 minutes. Despite detection of perineal erythema, swabs from days 3, 4, 5, 7, 10, and 12 were negative for HSV-2 by culture and by PCR for viral DNA.

### Previously infected C. apella monkeys are less susceptible to reinfection and shed low titers of HSV-2 after reinfection

**Fig 5:**
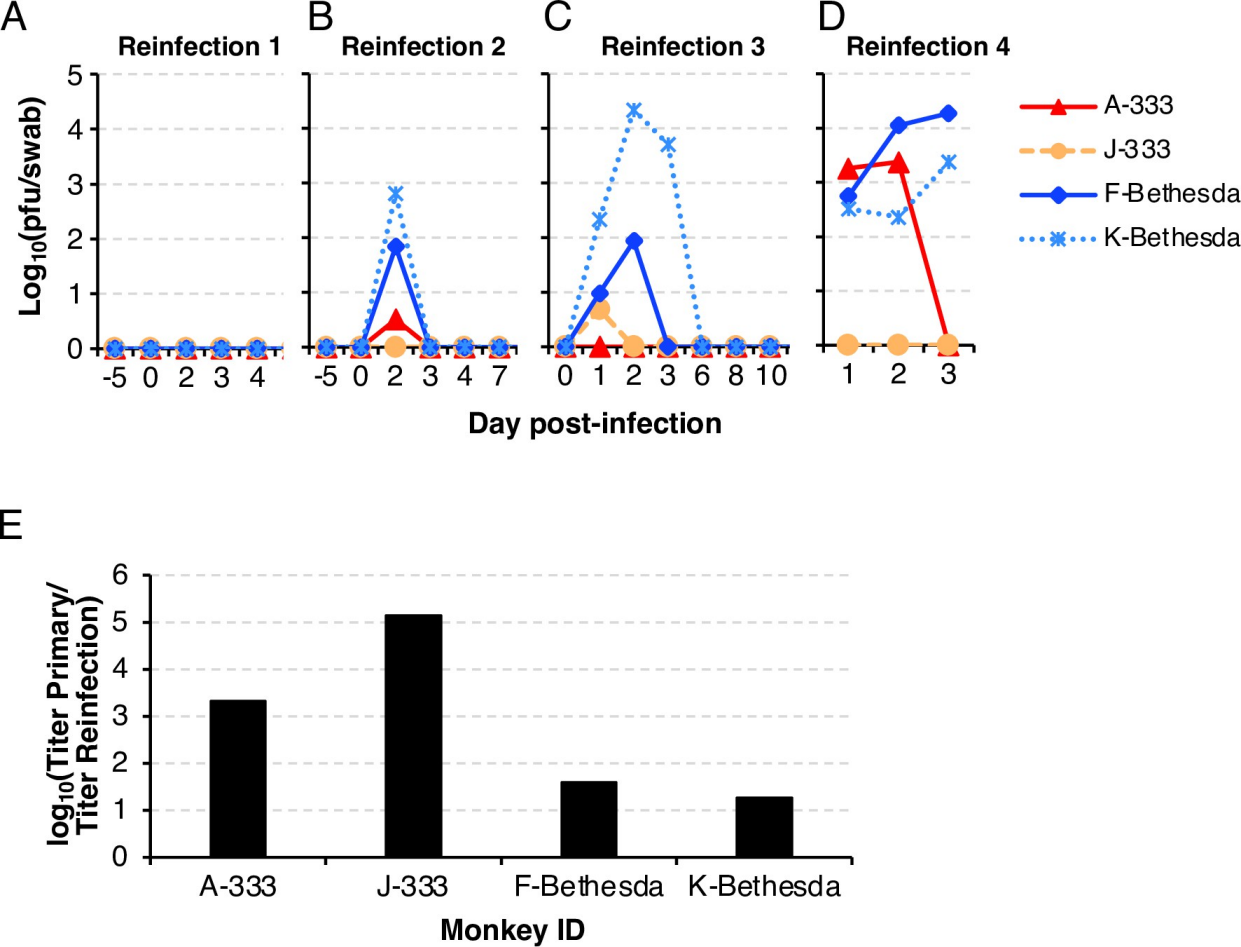
Previous intravaginal infection of C. apella monkeys reduced the severity of reinfection. Animals were sequentially re-infected on days 308, 378, 442, and 567 after primary infection, vaginal swabs were obtained and HSV-2 was titered in Vero cells. (A) First reinfection with 10^4^ pfu/monkey. (B) Second reinfection with 10^5^ pfu/monkey. (C) Third reinfection with 10^6^ pfu/monkey. (D) Fourth reinfection with 2 x 10^6^ pfu/monkey. (E) The ratio of the peak vaginal swab HSV-2 titer during primary infection to the highest peak swab titer from all the reinfections is shown. Data derived from **S4 Data**.

To determine if primary infection can reduce or prevent reinfection, monkeys were given a second dose of HSV-2 intravaginally. In order to distinguish latent viral DNA in dorsal root ganglia as a result of primary infection from subsequent infection with HSV-2, the virus strain used for reinfection was HSV-2 SD90e, a low passage clinical isolate from South Africa [32]. HSV-2 SD90e was given intravaginally to previously infected C. apella monkeys at a dose of 10^4^ pfu on day 308 after primary infection, and subsequent doses of 10^5^, 10^6^ and 2 x 10^6^ pfu were given intravaginally on days 378, 442, and 567, respectively. No lesions were observed after any of these doses of virus. No infectious virus was detected in the vaginal swabs after reinfection with the first dose of HSV SD-90e (10^4^ pfu, **Fig 5A**). Low titers of HSV-2 were only detected on day 2 from vaginal swabs after reinfection with the second dose of HSV-2 SD90e (10^5^ pfu). The two monkeys that had no lesions after primary infection, F-Bethesda and K-Bethesda, shed the highest titers of virus at 69 and 640 pfu/swab of HSV-2 SD90e, respectively, while the animals that had lesions after primary infection, A-333 and J-333 shed 3 pfu/swab and no detectable virus, respectively (**Fig 5B**). Animals receiving the third dose of HSV-2 SD90e (10^6^ pfu/monkey) had a similar pattern of shedding as those that received the second dose of virus; virus titers of swabs from monkeys F-Bethesda and K-Bethesda were higher (86 and 21,900 pfu/swab, respectively) than monkeys A-333 and J-333 (0 and 5 pfu/swab, respectively) (**Fig 5C**). The virus dose used for reinfection with the fourth dose of HSV2 SD90e (2x10^6^ pfu/monkey) was identical to the dose used for the primary infections. Vaginal swabs were collected on days 1, 2, and 3 after reinfection and monkeys were individually euthanized on days 3, 4, 5, and 6 to obtain ganglia for studies of latency and lymphoid tissues for T cell assays. The two monkeys that had no lesions after primary infection, F-Bethesda and K-Bethesda, shed 18,700 and 2,350 pfu/swab at their peak, respectively) (**Fig 5D**). One of the two animals that had lesions during primary infection (A-333) shed HSV-2 at a titer (1,810 pfu/swab and 2,370 pfu/swab) on day 1 and 2, respectively, that was comparable to that of monkeys F-Bethesda and K-Bethesda, but shedding was no longer detectable in monkey A-333 on day 3, while shedding increased in animals F-Bethesda and K-Bethesda on day 3. Another animal, J-333, that had lesions during primary infection had no detectable virus shedding on days 1, 2, and 3. Taken together, reinfection resulted in lower titers of HSV-2 shedding than that seen during primary infection, and titers after reinfection were especially low in the two monkeys that had vaginal lesions during primary infection. The peak shedding titers after reinfection of the animals that did not have lesions after primary infection were 18,700 and 21,900 pfu/swab (monkeys F-Bethesda and K-Bethesda, respectively), a 38- to 18-fold reduction compared with primary infection. The peak titers after reinfection for animals with lesions after primary infection were 2,370 and 5 pfu/swab (monkeys A-333 and J-333, respectively), a 2,140- and 140,000-fold reduction compared with primary infection (**Fig 5E**). These findings indicate that the animals developed a more protective immune response against re-infection when their primary infection was more severe.

### HSV-2 latent infection is detected in multiple ganglia of C. apella monkeys

All animals were euthanized after the fourth dose of HSV-2 SD90e and lumbar and sacral dorsal root ganglia, sacral sympathetic ganglia, and pelvic nerves were collected. DNA was extracted from these tissues and HSV-2 DNA was quantified by real-time qPCR. One set of PCR primers and probes (gGF2, gGR2, gGP2) were located in a region of the gG gene where the sequences of all three virus strains (333, Bethesda, and SD90e) are identical (**S1 Fig**). A second set of PCR primers and probes (UL39F1, UL39R1, UL39P1) were designed to distinguish HSV-2 333 and HSV-2 Bethesda from HSV-2 SD90e to differentiate primary infection from reinfection. Ten of 39 DNA samples from nervous system tissues were positive for HSV-2 DNA with copy numbers ranging from 2.2 to 169 copies/μg DNA extracted from the tissue, with the exception of dorsal root ganglion L5 from monkey F-Bethesda, that had a much higher viral DNA load of 619 copies/μg DNA (**Table 2**). HSV-2 SD90e DNA was only detectable in 1 of the 10 nervous system samples that were positive for HSV-2 when PCR was done with primers and probe for gG; dorsal root ganglion L5 from monkey F-Bethesda had 254 copies of HSV-2 SD90e/μg DNA. No viral DNA was detected in dorsal root ganglia above L4 or in sacral sympathetic ganglia. Interestingly, HSV-2 333 and HSV-2 Bethesda DNA were detected in pelvic nerves from 3 of 4 monkeys, although the copy numbers were low ranging from 6.5 to 22 copies/μg DNA. These results indicate that during primary infection of C. apella monkeys, HSV-2 infected the dorsal root ganglia and established a latent infection, although the genome copy numbers were lower than what has been observed in guinea pigs that have frequent recurrent lesions [33,34]. This low level of latent virus DNA in dorsal root ganglia may help to partially explain why recurrent genital lesions and recurrent virus shedding were infrequent. In addition, the only animal in which HSV-2 SD90e was detected in ganglia was monkey F-Bethesda which was euthanized 6 days after the fourth dose of HSV-2 SD90e infection and its vaginal swab titer peaked at day 3 with 18,720 copies/swab. Thus, the HSV-2 SD90e DNA detected in this dorsal root ganglion may have been from virus during the final reinfection rather than latent virus.

**Table 2:** HSV-2 viral DNA load in individual dorsal root and sacral sympathetic ganglia and pelvic nerve.

Primary infection strain	HSV-2 333				HSV-2 Bethesda			
Re-infection strain	SD90e				SD90e			
Animal ID	A-333		J-333		F-Bethesda		K-Bethesda	
PCR primers and probe specific for^a^	All three strains	SD90e only	All three strains	SD90e only	All three strains	SD90e only	All three strains	SD90e only
L1 DRG^**b**^	0^**c**^	0	0	0	0	0	0	0
L2 DRG	0	**−** ^**d**^	0	**−**	0	**−**	0	**−**
L3 DRG	0	**−**	0	**−**	0	**−**	0	**−**
L4 DRG	0	**−**	0	**−**	2.2	0	0	**−**
L5 DRG	0	**−**	0	**−**	619	254	0	**−**
S1 DRG	0	**−**	160	0	NTS^**e**^	NTS	23	0
S2 DRG	68	0	0	**−**	0	**−**	0	**−**
S3 DRG	43	0	0	**−**	0	**−**	73	0
Sacral Sympathetic G.	0	**−**	0	**−**	0	**−**	0	**−**
Pelvic Nerve	6.5	0	22	0	22	0	0	**−**

^**a**^ The primers and probe used to amplify all three HSV-2 strains correspond to a sequence located in HSV-2 gG; the primers and probe that only amplify HSV-2 SD90e correspond to a sequence located in HSV-2 UL39.

^**b**^ Dorsal root ganglion, L1 to L5 are lumbar ganglia and S1 to S3 are sacral ganglia.

^**c**^ Numbers indicate copies of HSV-2 DNA per **μ**g of total DNA extracted from the tissue.

^**d**^ “-” indicates test not done.

^**e**^ NTS indicates no tissue sample available.

### HSV-2 intravaginal infection induces serum neutralizing antibody responses in C. apella monkeys

**Fig 6:**
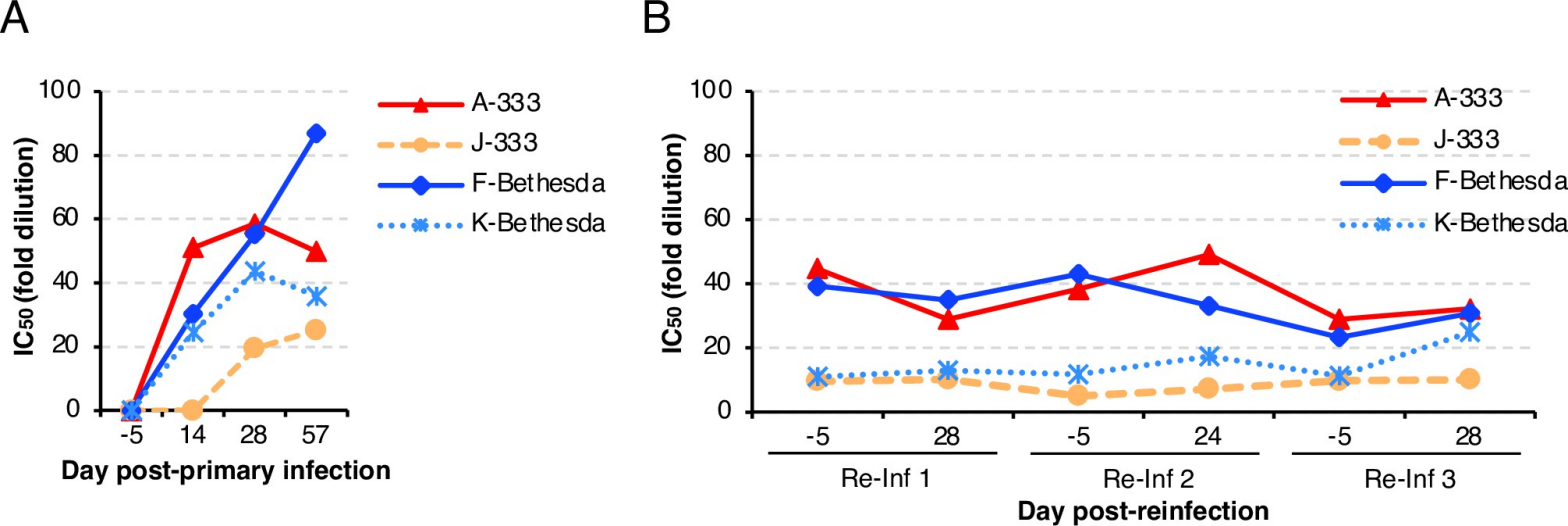
Serum HSV-2 neutralizing titers of C. apella monkeys after primary infection and reinfection with HSV-2. Blood was collected before and after primary infection (A) or after reinfection (B). After heat-inactivation of sera, HSV-2 neutralizing titers were determined in Vero cells. The IC_50_ was calculated by non-linear regression with Prism 8 software and shown as the reciprocal of the serum dilution. Data derived from **S5 Data**.

Serum HSV-2 neutralizing titers were determined using Vero cells. Serum neutralizing activity could be detected 14 days post-primary infection. The neutralizing titers in 2 (A-333 and K-Bethesda) out of 4 monkeys peaked on day 28 (IC_50_ of 58.6 and 43.7, respectively), while the titers in the other two monkeys (F-Bethesda and J-333) peaked on day 57 (IC_50_ of 87.0 and 25.3, respectively) (**Fig 6A**). The serum neutralizing titer changed very little after reinfections (**Fig 6B**) ranging from 0.6 to 2.2-fold at 28 days after reinfection compared to 5 days prior to reinfection. The change averaged 0.9-fold after the first reinfection, 1.2-fold after the second reinfection, and 1.4-fold after the third reinfection.

### HSV-2 intravaginal infection induces virus-specific T cell responses in peripheral blood, spleen, and lymph nodes in C. apella monkeys

**Fig 7:**
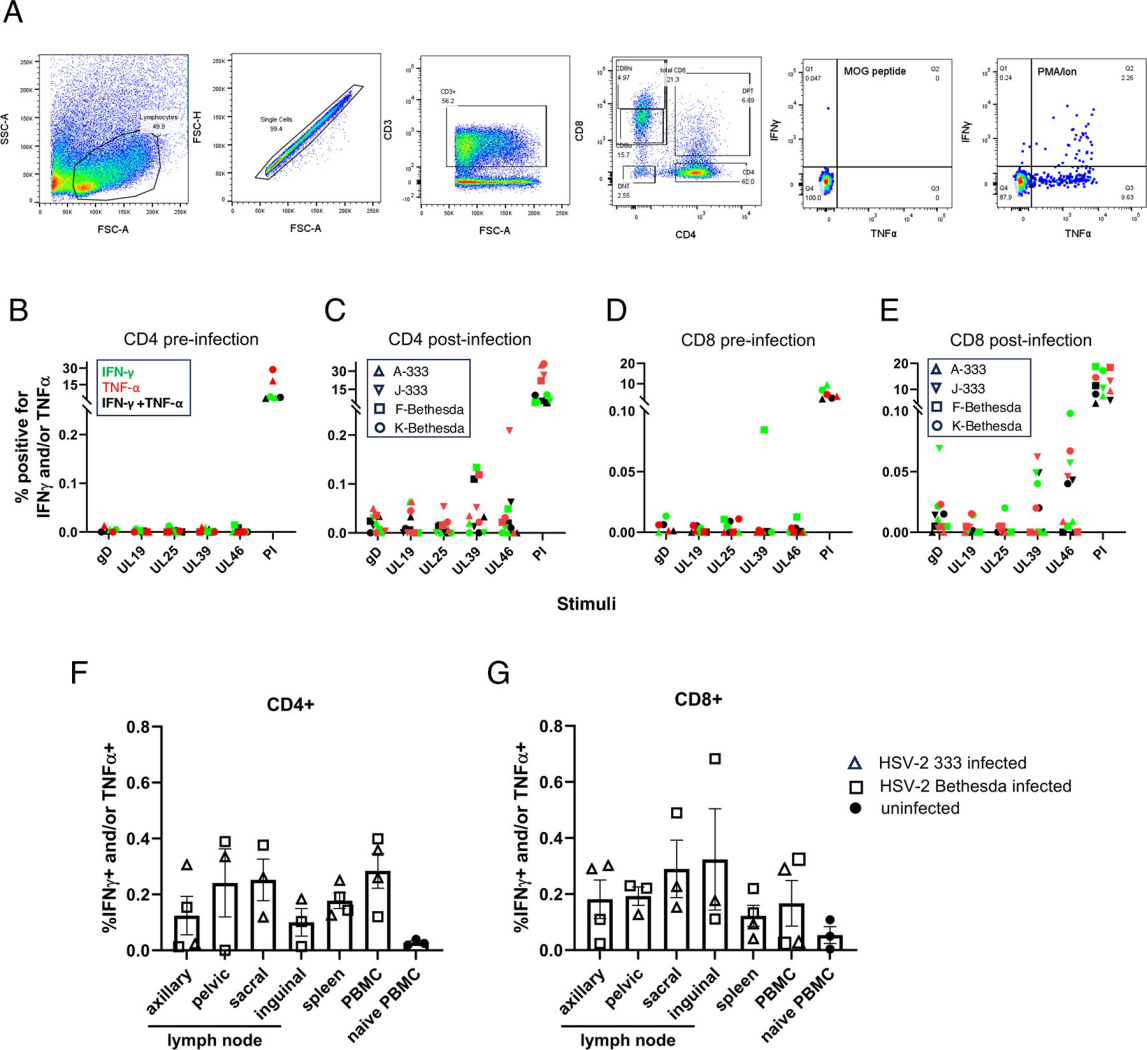
Cellular immune responses to HSV-2 in C. apella monkeys. Cryopreserved PBMCs and dissociated cells from lymph nodes and spleen were thawed and rested overnight and then stimulated for 8 hours with HSV-2 gD, U19, UL25, UL39, or UL46 peptide pools at a concentration of 1 ug/ml for each peptide. Cells were stained with anti-CD3-BV421, anti-CD4-BV650, and anti-CD8-PE, permeabilized, stained intracellularly for TNF-α and IFN-γ, and examined by flow cytometry. (A) Example of gating of lymphocytes from deep inguinal lymph nodes showing T cell populations and the results of TNF-α and IFN-γ staining of CD8+ cells after incubation with negative (MOG peptide) and positive (PMA/ionomycin) control stimuli. In the remaining panels, the data shown are net positive percent of cells, in which the percent of cells that were positive for MOG peptide (the negative control) was subtracted from the percent of cells positive for the cytokines after HSV-2 stimuli. (B,D) PBMCs from uninfected C. apella monkeys (not from the four monkeys in panels C and E) and (C, E) from infected monkeys stimulated with HSV-2 peptide pools or phorbol myristate acetate plus ionomycin (PI) as indicated on the x axis, were gated for CD4 (B,C) and CD8 (D,E) and IFN-γ and TNF-α. (F, G). Cells from lymph nodes, spleen, or PBMCs from HSV-2 infected C. apella monkeys (or naïve PBMCs from uninfected monkeys) were stained as described in panels B-E and the percentage of IFN-γ and/or TNF-α positive cells after each peptide pool stimulation were combined for each organ as indicated on the x axis; the results from CD4 or CD8 cells are shown in (F) or (G). FSC-A, forward scatter parameter; FSC-H, forward scatter height; SSC-A, side scatter parameter. Data derived from **S6 Data**.

To study the cellular immune to HSV-2 infection in C. apella monkeys, animals were euthanized 3–6 days after the fourth reinfection and spleen, and axillary, deep inguinal, pelvic, and sacral lymph nodes were harvested and PBMCs were obtained from peripheral blood. Cells were stimulated with HSV-2 peptide pools from gD, UL19 (major capsid protein), UL25 (auxiliary capsid protein), UL39 (large subunit of ribonucleotide reductase), and UL46 (tegument protein). Cells were then stained for T cell markers CD3, CD4, and CD8 and then permeabilized and stained for intracellular IFN-γ and TNF-α (**Fig 7A**). The net positive percent of cells that were IFN-γ and/or TNF-α positive was determined by subtracting the percent of the cells that were positive for myelin oligodendrocyte glycoprotein peptide (MOG, the negative control) from the cells positive for IFN-γ and/or TNF-α.

CD4 (**Fig 7B**) and CD8 (**Fig 7D**) T cells from the peripheral blood of uninfected C. apella monkeys had very low responses to stimulation with the HSV-2 peptide pools, indicating the low background of the assay. Both CD4 (**Fig 7C**) and CD8 (**Fig 7E**) T cell responses to the HSV-2 peptide pools were detected in peripheral blood from all four animals. Peptides from UL39 were the most prominent targets of CD4 cells and peptides from UL39 and UL46 were most often targeted by CD8 cells in the peripheral blood of the animals. CD4 cells expressing TNF-α were more prominent than those expressing IFN-γ in spleen; some CD4 and CD8 cells expressed both IFN-γ and TNF-α.

When lymph nodes at different sites were compared, there was a tendency for higher numbers of HSV-2-specific CD4 cells expressing IFN-γ and/or TNF-α in the pelvic and sacral lymph nodes than the axillary nodes (**Fig 7F**), and higher numbers of HSV-2-specific CD8 cells expressing either or both of the cytokines in the sacral or deep inguinal nodes than in the axillary nodes **(Fig 7G**). Thus, there tended to be higher numbers of HSV-2-specific T cells expressing cytokines in lymph nodes closer to the site of infection.

In the spleen, more of the HSV-2-specific CD4 and CD8 cells expressed IFN-γ than TNF-α and few expressed both cytokines (**S2 Fig**). Similarly, more HSV-2 specific CD4 and CD8 cells from axillary lymph nodes expressed IFN-γ than TNF-α (**S3 Fig**). On the other hand, HSV-2-specific T cells from pelvic and deep inguinal lymph nodes had similar numbers of cells expressing TNF-α or IFN-γ (**S3 Fig**).

In contrast to humans, C. apella monkey T cells had large populations of CD4+CD8+ (double positive) T cells that recognized HSV-2 peptides, particularly for UL39 peptides in sacral lymph nodes (**S2 and S3 Figs**). Double positive T cells are found at increased frequency in humans with certain viral infections [35]. C. apella uninfected monkeys also had higher levels of CD4+CD8+ cells.

### HSV-2 grows less well in C. apella primary fibroblasts than it does in human primary fibroblasts but much better than in rhesus macaque fibroblasts

**Fig 8:**
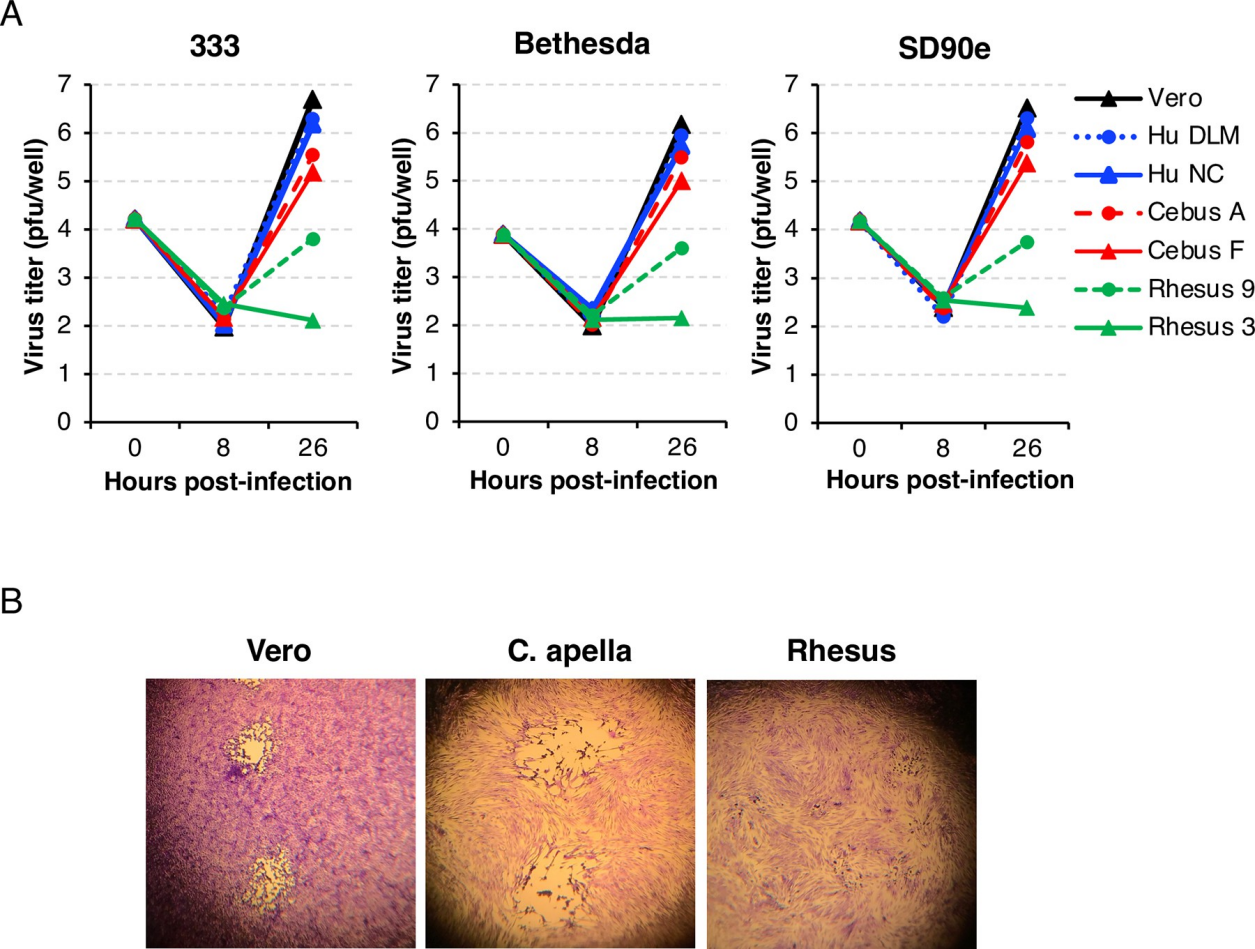
HSV-2 grows to higher titers in C. apella primary fibroblasts than in rhesus macaque primary fibroblasts. (A) Growth curve of HSV-2 strains 333, Bethesda, and SD90e in Vero cells and in human, C. apella, and rhesus macaque primary fibroblasts. Cells were infected with virus at a multiplicity of infection of 0.1 (based on HSV-2 titrated on Vero cells). (B) plaque size of HSV-2 strain 333 on Vero cells, and C. apella and rhesus macaque primary fibroblasts. Data derived from **S9 Data**.

In contrast to humans and C. apella monkeys, only a minority of rhesus monkeys infected with HSV-2 shed infectious virus and none develop lesions [25]. To better understand why C. apella monkeys might be a better model for HSV-2 infection compared with rhesus macaques, we studied the three HSV-2 strains used in our animal study for their ability to replicate in primary fibroblasts from C. apella, rhesus macaque, and humans. Two primary fibroblast lines from each species were infected with HSV-2 strain 333, Bethesda, or SD90e at a multiplicity of infection of 0.1 and cultures were harvested at 8 and 26 hours after infection. Both the infected cells and the cell culture supernatant were harvested together, subjected to 3 cycles of freezing and thawing, centrifuged, and the resulting supernatant containing cell-free virus used for titration on Vero cells. The fold-increase in the infectious virus yield at 26 hours after infection compared to the input virus ranged from 190–300, 91–106, 15–29, and 0.19–0.27 in Vero cells, human fibroblasts, C. apella fibroblasts, and rhesus fibroblasts, respectively (**Fig 8A**). At 24 hours after infection, the virus yield from rhesus primary fibroblasts was less than the input virus, while HSV-2 replication in C. apella primary fibroblasts was about 17% of that in human primary fibroblasts. HSV-2 (333) produced large plaques on Vero cells and C. apella fibroblast monolayers, but only resulted in small foci of cytopathic effects and no plaques on rhesus fibroblasts (**Fig 8B**).

## Discussion

We found that C. apella monkeys inoculated intravaginally with HSV-2 strain 333 or HSV-2 Bethesda shed infectious virus from the vagina for at least 7 to 9 days. Monkeys infected with HSV-2 333 developed vesicular vaginal lesions, while those infected with HSV-2 Bethesda did not have visible lesions. Therefore, infection of C. apella monkeys with HSV-2 Bethesda more closely resembles HSV-2 primary infection in humans in that most humans have asymptomatic primary infections [36] Shedding of HSV-2 DNA in C. apella monkeys, based on the qPCR assay, persisted for 7 to 10 days after infectious virus was no longer detectable. Infectious virus could be detected from all swabs when the HSV-2 DNA copy number per swab was ≥ 1.38x10^5^, while no infectious virus could be detected when the copy number was ≤ 3.78x10^4^. In a study of human genital swabs, HSV DNA was detected in amounts ≥ 10^4^ copies/swab; infectious virus was detectable in 45% of the samples, compared with only 6% of samples with fewer copies of HSV DNA [37]. Thus, the level of HSV-2 DNA copies/swab reflected the ability to detect infectious virus shed from both C. apella monkeys and humans.

None of the infected C. apella monkeys had neurologic complications (such as urinary retention, hind limb paralysis) unlike what is observed after intravaginal infection of mice and guinea pigs [15,29]. These findings are similar to those reported by others who studied C. albifrons [22,23], or C. apella [24] monkeys. This reiterates our observations that primary infection of C. apella monkeys more closely recapitulates the course of the disease in humans than in mice and guinea pigs.

HSV-2 DNA was detected in plasma, but not in PBMCs in all 4 C. apella monkeys shortly after they were initally infected intravaginally. In a study of women with primary genital herpes, about 24% had HSV DNA detected in their plasma; the highest positive rate was during the first week of symptoms [38]. The higher percentage (100%) of plasma HSV-2 DNA positivity in our C. apella monkeys was likely due to our ability to sample monkeys before onset of symptoms at the time when the level of HSV-2 DNA peaked in the plasma; in humans obtaining samples at such time points is virtually impossible. We detected HSV-2 DNA in plasma, but not in PBMCs of C. apella monkeys, similar to that reported in a study that detected HSV-2 DNA in human plasma but not PBMCs from persons with primary genital herpes [39]. In contrast, intravaginal infection of mice with HSV-2 showed that infectious HSV-2 was present predominantly in platelets rather than in white blood cells or serum [40]. Thus, detection of HSV DNA in the plasma of C. apella monkeys more closely resembles what occurs in humans than in mice.

Despite detecting high titers of HSV-2 in vaginal swabs during primary infection indicative of robust virus replication, we did not observe frequent spontaneous reactivation of HSV-2 or reactivation after UV exposure. Latent HSV-2 DNA was however detected in sacral dorsal root ganglia (DRG) in 3 of the 4 animals and in lumbar DRG in the fourth animal (**Table 2**). Detection of latent HSV-2 viral DNA in DRG of C. apella has not been previously reported. A prior study in C. albifrons monkeys over 40 years ago reported recovery of HSV-2 virus from sacral DRG 7 or 43 days after infection [41]. The mean latent viral DNA load in DRG from our C. apella monkeys that were HSV-2 DNA positive was 78.5 copies/ug DNA, which was much lower than that reported in guinea pigs with mean copy numbers of 430 copies/ug DNA [42], 850 copies/ug DNA [34], and 10,150 copies/ug DNA [33]. We have shown that a higher HSV-2 DNA load in ganglia correlates with increased virus reactivation in mice and guinea pigs [34,43]. The relatively low level of latent viral DNA in the DRG of C. apella monkeys may in part be responsible for the very low level of spontaneous reactivation and lack of reactivation after UV exposure. The absence of recurrent lesions and very rare instances of virus shedding after the acute phase of infection indicates that the C. apella monkey genital herpes model is not suitable for evaluation of therapeutic vaccines.

We and others have found that cebus monkeys develop vaginal lesions after HSV-2 intravaginal infection without other symptoms [22,23,27]. Other studies showed that owl monkeys, another New World monkey, usually develop fatal disease after HSV infection [26]. In contrast, Old World monkeys including rhesus macaques and baboons do not develop lesions after HSV-2 infection [23,25]. Thus, in general, New World monkeys infected with HSV-2 have more severe disease than Old World monkeys. The exception is squirrel monkeys, which are New World monkeys that do not develop disease after HSV infection [23]. HSV-2 is thought to have originated in Africa and spread worldwide with the migration of humans out of Africa [44]. These findings are consistent with the much higher frequency of HSV-2 in Africa than on other continents [45]. Thus, HSV-2 presumably has had a longer time to evolve with Old World primates than with New World primates and therefore the disease may be milder in Old World primates.

We found HSV-2 neutralizing titers in the serum of C. apella monkeys 14 days after primary infection, similar to humans who develop neutralizing titers about 2 weeks after primary infection [46]. The neutralizing titer changed very little after reinfection. In a prior study of rhesus macaques infected intravaginally with HSV-2, the animals developed HSV-2 peptide-specific T cells but not HSV-2 antibody responses [25]. In contrast, C. apella monkeys in our study developed both B cell and T cell responses to HSV-2.

The most prominent targets of CD4 cells in the blood of the C. apella monkeys were peptides corresponding to HSV UL39, and the most common targets for CD8 cells were peptides corresponding to UL39 and UL46. Hosken and colleagues [47] found that UL39 was the most frequent target of HSV-2 specific CD8 cells in humans and that UL46 was among the top 5 viral proteins recognized by CD8 cells. We found that UL39 and UL46 were also prominent targets of CD4 cells in the lymph nodes of C. apella monkeys. A study of CD4 T cells in the cervix of humans infected with HSV-2 reported that UL39 and UL46 were among the most common targets of CD4 T cells [48].

We found HSV-2-specific CD4 and CD8 cells expressing IFN-γ and/or TNF-α in pelvic, sacral, and inguinal lymph nodes of C. apella monkeys infected with HSV-2. IFN-γ has a key role in controlling HSV infection in vitro [49], in mice [50], and in humans [51]. TNF-α is important for controlling HSV-2 infection in mice [52] and likely in humans [53] and is synergistic with IFN-γ to block HSV replication in vitro [54].

HSV-2 is thought to have been transmitted from a nonhuman primate to a precursor of humans [20]; thus, HSV-2 proteins have coevolved with primates and many of the viral proteins, including immune evasion molecules which have less or no activity in mice and guinea pigs [55, 56] are more likely to be active in nonhuman primates; thus these animals may serve as better models than mice or guinea pigs to predict the efficacy of vaccines in humans.

Lo and colleagues recently reported a study in which rhesus monkeys were infected intravaginally with HSV-2 at multiple short intervals [25]. While a minority of rhesus monkeys shed infectious HSV-2 for a few days (virus titers were not reported), we found that all of our C. apella monkeys shed high titer of infectious virus for at least 7 days after a single dose of HSV-2. None of the rhesus monkeys had vaginal lesions after repeated intravaginal dosing; however, 2 of 4 C. apella monkeys (both of which received HSV-2 333) developed vaginal lesions during primary HSV-2 infection. While rhesus monkeys were reported to reactivate HSV-2 detected by PCR of vaginal swabs, nearly all the positive samples were collected 14–28 days after the last inoculation. In contrast, all of our C. apella monkeys shed virus for at least 7–11 days after infection and viral DNA shedding did not stop until day 21 to day 28 after infection; viral DNA was only detected twice thereafter and at very low levels. The sacral ganglia are the main sites of latency of HSV-2 in humans [57], mice [16], and guinea pigs [15] after intravaginal infection. In the rhesus monkey study [25], latent HSV-2 DNA was not detected in sacral ganglia of the animals after infection, while viral DNA was frequently detected in the sacral ganglia of our C. apella monkeys. Most rhesus monkeys did not have antibody responses to HSV-2; in contrast, all C. apella monkeys in our study developed neutralizing antibody to HSV-2. Thus, infection of C. apella with HSV-2 is a better animal model than rhesus macaques for recapitulating the course of acute infection of humans with HSV-2. One possible explanation for this is that cells from C. apella can better support HSV-2 replication than cells from rhesus macaques. We found that 26 hours after HSV-2 infection of primary fibroblasts from C. apella there was a 15–29 fold increase in virus titer compared with input virus, while infection of primary fibroblasts from rhesus macaques did not show an increase in virus titer over the input inoculum. A prior study showed that HSV-1 and HSV-2 had 100-fold lower yields in rhesus macaque fibroblasts compared with HeLa cells [58].

Other viral infections have different pathogenesis depending on the species of primate tested. Simian immunodeficiency virus replicates to high levels in sooty mangabeys and African green monkeys and generally does not cause immunodeficiency; however, the same virus causes an AIDS-like disease in macaques [59]. Simian varicella virus (SVV) infects rhesus monkeys in which it causes a varicella-like syndrome followed by establishment of latency; it can subsequently reactivate [60]. In contrast, SVV infection of African green monkeys causes a persistent infection with continued viral transcription in tissues for months after infection [61]. B virus, a close homolog of HSV, usually is asymptomatic in rhesus macaques, but can cause a fatal encephalitis in humans [62].

The C. apella model does have several limitations. There are no established large breeding colonies of animals commercially available. Reagents for immunological studies of these animals are very limited. Spontaneous reactivation of HSV-2 is rarely detected in C. apella monkeys and we were unable to induce reactivation in the animals with UV radiation. Thus, the C. apella model would not be appropriate for testing therapeutic vaccines.

In summary, the C. apella monkey model provides a number of advantages over the current animal models being used to study prophylactic vaccines for genital herpes. Unlike mice and guinea pigs, the most common models for HSV-2, but similar to humans, C. apella monkeys do not develop neurologic disease after intravaginal inoculation. The presence of multiple, persistent lesions in C. apella monkeys provides an opportunity for evaluating the ability of a prophylactic HSV-2 vaccine to reduce the number of lesions and their duration. Persistent, high titer vaginal shedding of infectious virus allows the ability to evaluate prophylactic vaccines to reduce virus shedding and potentially reduce transmission after virus challenge. The presence of antibody to HSV-2 in the serum and T cell responses to HSV-2 antigens in circulating T cells allows immune monitoring and the possibility of identifying immune correlates of protection from infection in this model. Thus, intravaginal infection of C. apella monkeys with HSV-2 may serve as an improved model to evaluate prophylactic vaccine candidates, antivirals and monoclonal antibodies to prevent infection or reduce disease associated with primary HSV-2 infection before such measures are tested in humans.

## Materials and methods

### Ethics statement

All monkey studies were conducted under a protocol approved by the Animal Care and Use Committee of the National Institute of Allergy and Infectious Diseases and the studies were carried out in strict accordance with the recommendations in the Guide for the Care and Use of Laboratory Animals of the National Institutes of Health.

### Cells and virus

Vero cells were purchased from ATCC and maintained in DMEM (Gibco 11960–044) with 10% fetal bovine serum (FBS), 1x penicillin/streptomycin/glutamine (Gibco 10378–016). HSV-2 strain 333, a gift from Dr. Gary Hayward at Johns Hopkins University, was subjected to 3 cycles of plaque purification and a non-syncytial clone R519 was obtained and propagated in Vero cells. The virulence of the R519 clone of HSV-2 333 has been documented in mice [10, 63]. HSV-2 strain Bethesda was a clinical isolate passed 3 times in human fibroblast MRC5 cells [64]. HSV-2 Bethesda generates relatively small plaques indicating that it does not spread rapidly from cell to cell. SD90e was a low passage of clinical isolate [64], a gift from David Knipe (Harvard Medical School). The genomes of all the three strains have been sequenced [64]. Cell-free viruses used in this project were all propagated in Vero cell cultures, aliquoted, and stored at -80°C. The titrations of the virus stocks were performed in Vero cell cultures. Inocula for primary infection and later dose-escalated repeated infection were diluted from the stocks to the desired concentration with Vero cell culture medium and stored at -80°C.

### Monkey studies

Female C. apella monkeys (1 to 2.6 years old at the time of primary infection) were purchased from AlphaGenesis. Animals were screened for neutralizing antibody to HSV-2 before infection and none of the animals had antibody. Monkeys were sedated with 10–20 mg/kg of ketamine before procedures.

Five days before HSV-2 infection (day -5), 20 mg of Depo-Provera (Pfizer, 0009-0626-01) diluted in Dulbecco’s Phosphate Buffered Saline (DPBS) (Gibco 14190–144) were given to monkeys intramuscularly. On the day of primary infection, 2x10^6^ pfu of stock HSV-2 333 or HSV-2 Bethesda in 50 ul of volume was instilled into the vaginal vault using a P200 pipette tip. The animals were held upside down for 5 minutes and then laid flat until they recovered from sedation.

Vaginal swab samples were taken on desired days with a polyester tipped applicator (Puritan Medical Products Co, 25–801 D 50). Swabs to be used later for virus cultures were stored in vials with 0.7 ml of swab storage medium (DMEM with 10% FBS, 1x penicillin/streptomycin/glutamine, 1 x amphotericin B). Swabs to be used later for PCR were stored in vials with 0.5 ml of DPBS; all swab samples were frozen on dry ice and stored at -80°C. When lesions were observed, their surface was swabbed for virus culture and PCR for HSV-2 DNA.

On the days of blood collection as shown in Table 1, blood was drawn to obtain serum or plasma and PBMCs were isolated using Ficoll density gradient centrifugation. Serum and plasma samples were stored in -80°C and PBMCs were stored in a liquid nitrogen freezer.

Systemic observation of the health of monkeys included body weight, temperature, appetite, and integrity of skin. Genital lesions were scored as follows: normal as 0, redness as 0.5; one small lesion as 1; one medium lesion as 2; one large lesion or ulcer as 3. If multiple lesions presented simultaneously, the score was the sum of each lesion score.

Ultraviolet (UV) light irradiation of monkey perineum was first performed on day 214 after primary infection. After sedation of the animals, the perineum was exposed to a UV source (UVP Transilluminator T-20, λ = 302, 600 mJ/cm^2^/min) through a 3 cm x 3cm window for 2 minutes. A second treatment was done on day 235 post-primary infection and the exposure time was increased to 4 minutes. Vaginal swabs were obtained immediately before the UV irradiation, and on days 3, 4, 5, 7 after each irradiation and cultured for infectious virus in Vero cells and assayed for viral DNA by real-time PCR.

Intravaginal reinfection of the monkeys was done as reported above for primary infection, but with different doses of HSV-2 strain SD90e virus. Five days before each reinfection, monkeys were injected with Depo-Provera. Reinfection was first performed with a dose of 10^4^ pfu/monkey on day 308 post-primary infection, and subsequent doses of 10^5^, 10^6^, and 2 x 10^6^ pfu/monkey were given intravaginally on days 378, 442, and 567, respectively. Vaginal swabs were obtained up to 7–10 days after infection for virus cultures. Blood was collected on day -5 and about 4 weeks after reinfections to determine the serum neutralizing titers.

C. apella monkeys K-Bethesda, J-333, F-Bethesda, and A-333 were terminally bled under sedation to obtain plasma and PBMCs and then euthanized and necropsied on days 3, 4, 5, and 6 after the fourth reinfection, respectively. Spleens and lymph nodes were collected and minced in RPMI 1640 with 5% FBS to dissociate the lymphocytes. Cell pellets were resuspended in RPMI 1640 with 20% FBS for cryo-preservation. Lumbar and sacral DRGs and pelvic nerves were collected from right side of body were snap frozen on dry ice and stored at -80°C.

### Virus titration of swab samples (plaque assays)

Swab samples were thawed on ice, vortexed, briefly centrifugated, and supernatants were serial diluted 10-fold starting from 1:2. 0.5 ml of diluted samples were added to each well in 6-well plates that had Vero cell monolayers. After incubation for one hour at 37°C in a CO_2_ incubator, the inoculum was removed, and 2.5 ml of swab titration overlay medium was added. The overlay medium was Vero cell culture medium supplemented with 1x amphotericin B and 0.5% of Gamunex-C (10% human gamma-globulin intravenous injection solution, Talecris Biotherapeutics). Cells were stained 2 days later with plaque staining solution (10% formaldehyde, 5% acetic acid, 60% methanol, 1% W/V crystal violet) and plaques were counted under a microscope. The virus titer was calculated as the pfu/swab.

### HSV-2 growth in primary fibroblasts

Muscles were obtained at necropsy from 2 C. apella monkeys and 2 rhesus macaques. The tissues were minced and cultured with fibroblast growth medium (MEM alpha medium supplemented with10% FBS, 1x penicillin/streptomycin/glutamine, and 0.2% primocin which contains four compounds that are active against mycoplasma, bacteria, and fungi) at 37°C in a CO_2_ incubator. Primary fibroblasts cultured from human skin biopsies were from healthy volunteers who signed a consent on an NIH Internal Review Board Protocol. To examine the ability of the primate primary fibroblasts and Vero cells to support HSV-2 replication, 1.1 x 10^5^ fibroblasts were seeded into each well of 12-well plates, and the next day the cells were nearly confluent and infected with HSV-2 strain 333, Bethesda, or SD90e at a multiplicity of infection of 0.1 (titrated on Vero cells). Infected cultures were harvested at 8 and 26 hours after infection, cell-free virus was prepared, and virus titers were performed on Vero cells.

### DNA extractions

DNA was isolated using a QIAgen DNeasy Blood and Tissue Kit (QIAgen 69504) with some modifications. Swab samples or plasma samples for PCR were thawed on ice and vortexed. After every 4 samples a DPBS sample was inserted to monitor for contamination as a negative control for DNA extraction and qPCR. 200 ul of swab sample or negative control was mixed with 200 ul of AL buffer from the kit, 1 ul of 4 ug/ul carrier RNA (QIAgen), 1 ul of 20,000 copies/ul DNA of plasmid containing the human VR1 gene segment (to serve as a DNA extraction efficiency control), and 20 ul of proteinase K. The mixtures were vortexed and incubated at 56°C in a water bath with shaking for 1 hour; the rest of the steps followed the standard protocol from the manufacturer. DNA was eluted from the column with 100 ul of AE buffer. For plasma samples from day 7, 9, 11, and 14, only 100 ul of plasma was used and DNA was eluted in 50 ul of AE buffer. DNA samples were stored at -20°C. To extract DNA from PBMCs, about 1.7x10^6^ cells from 0.5 ml of blood were resuspended in 200 ul of DPBS, and the rest of the steps were identical to that described above without the addition of carrier RNA; DNA was eluted from the column with 200 ul of AE buffer. To extract DNA from dorsal root ganglia, the tissues were minced with a scalpel and resuspended in 360 ul of ATL buffer and 40 ul of proteinase K (if the tissue was >25 mg, the amounts of ATL and proteinase K were doubled). A vial contained ATL/proteinase K was inserted to monitor contamination after each 10 sample vials. The vials were incubated in a 56°C shaking water bath overnight. Each tissue lysate was divided into 2 tubes and the rest of the steps followed the instruction from the kit; DNA eluted with 200 ul of AE buffer from each column. The concentration of DNA was calculated based on reading absorbance at 260 nm.

### Serum HSV-2 neutralizing titers

Serum samples were heat inactivated at 56°C for 30 minutes, cooled to room temperature, and were serially diluted (2-fold dilutions) with 2% FBS in DPBS in 48-well plates. 150 ul of diluted serum was mixed with 150 ul of HSV-2 333 clone R519 (containing about 60–80 pfu) and incubated at room temperature for one hour. The diluted serum and virus mixture was then added to Vero cell monolayers in 12-well plates and the remaining steps were performed as described above for plaque assays. Two pre-infection serum samples were included with each neutralization assay. The concentration of sera that inhibited plaque formation by 50% (IC_50_) was determined using the non-linear regression function of Prism 9 software and expressed as the reciprocal of the serum dilution.

### Quantification of HSV-2 DNA in swabs, plasma, and PBMCs

The amount of HSV-2 DNA on vaginal and lesion swabs and in plasma and PBMCs was determined by TaqMan real time qPCR with primers and probe specific for the HSV-2 gG gene. Each 25 ul PCR reaction contained 10 ul of sample DNA, 12.5 ul of 2x TaqMan Universal PCR Master Mix (ThermoFisher, 4304437), 1 uM each of primers gG2F2 (5’ GCT CCC GCT AAG GAC ATG C 3’) and gG2R2 (5’ GAT GAT AAA GAG GAT ATC TAG AGC AGG G 3’), 200 nM of probe gG2P2 (5’FAM-TCC CCC TGT TCT GGT TCC TAA CGG C-TAMRA3’), 0.5% BSA, 1,000 copies of plasmid DNA containing the mouse TimP3 sequence as an amplification efficiency standard and mouse TimP3 primers and probe mix (Life Technologies, Assay ID Mm00441826_m1). The qPCR reactions were performed in a QuantStudio 3 instrument (ThermoFisher, A28137) for 40 standard thermal cycles with the exception of denaturing at 95°C for 20 seconds. Based on the total amount of DNA from each swab, 200 ul plasma, and the number of PBMCs, HSV-2 DNA copies in these samples were expressed as copies/swab, copies/ml plasma, and copies/10^6^ cells, respectively. None of the negative controls (DPBS or ATL buffer/proteinase K only vials) had detectable HSV-2 DNA by qPCR.

### HSV-2 DNA load in dorsal root ganglia and pelvic nerves

gG specific primers and probe were used to quantitate viral DNA (200 to 500 ng/reaction) from all three HSV-2 strains: 333, Bethesda, and SD90e. To determine if the source of viral DNA in ganglia was from primary infection versus reinfection, an SD90e UL39 specific primer and probe set was used: SD90eUL39F1 (5’ CGC GAT CTC AAA CGT CGG 3’), SDeLU39R1 (5’ GTC CTT GGG TTC CCG TG 3’), and SD90eUL39P1 (5’ 6-FAM-ACC TCC GCG /ZEN/ ACT ACA TCC GTG-IABkFQ 3’). These primers and probe amplify HSV-2 SD90e DNA, but not HSV-2 333 or HSV-2 Bethesda DNA **(S1 Fig)**. qPCR conditions were similar to those used in the section above “Quantification of HSV-2 DNA in swabs, plasma, and PBMCs.”

### HSV-2 specific T cell responses in C. apella PBMCs, spleens and lymph nodes after the fourth reinfection

Frozen lymphocytes dissociated from spleens and lymph nodes, or from PBMCs were thawed, rested overnight at 2 x 10^6^ cells/mL in RPMI supplemented with 10% FBS, penicillin/streptomycin/glutamine, and 10 U/mL of human IL-2 (National Cancer Institute Biologics Repository). Cells were then stimulated for 8 hours with peptide pools containing 1ug/mL of each peptide of HSV-2 gD, UL19 upper domain, UL25, UL39 or UL46 in RPMI supplemented with 5% FBS, penicillin/streptomycin/glutamine at 2 x 10^6^ cells/mL in 96-well round bottom plates. Negative controls were 1% DMSO (the solvent used to dissolve the HSV peptides) and myelin oligodendrocyte glycoprotein (MOG) 35–55 peptide (1ug/mL, a peptide control); the positive control was 20ng/ml phorbol myristate acetate (Sigma) plus 1 ug/ml ionomycin (Sigma) (PI) stimulation for 6 hours. All wells were co-stimulated along with 1 ug/mL of antibody to human CD28 and human CD49d (BioLegend, clones CD28.2 and 9F10, respectively) except the PI wells which did not receive the costimulatory antibodies. After the first hour of stimulation, brefeldin A and monensin (BioLegend) were added to a final concentration of 5 ug/ml and 2 uM, respectively. During the last 30 minutes of stimulation, the cells were stained with anti-human CD3-BV421 (BD, clone Sp34-2), anti-human CD4-BV650 (BD, clone L200), and anti-marmoset CD8-PE (BioLegend, clone 6F10). The cells were washed and stained with near IR live/dead (Life Technologies) for 10 minutes at room temperature. The cells were then washed and fixed in 4% paraformaldehyde for 15 minutes at 37°C. The following morning the cells were washed and permeabilized/fixed with Cytofix/Cytoperm (BD Biosciences) for 25 minutes on ice. The cells were washed with Perm wash (BD Biosciences) and intracellular staining was performing using anti-human TNF-α-APC (MabTech, clone MT15B15) and anti-human IFN-γ -FITC (MabTech, clone 1-D1K) for 60 minutes at room temperature. The antibodies used for cell surface and intracellular staining were tested and found to recognize C. apella splenocytes. The cells were washed and flow cytometry was performed using an LSR Fortessa (BD Biosciences) and data were analyzed in FlowJo 10.

## Supporting information

S1 Fig PCR primers and probes amplify all three HSV-2 strains and distinguish SD90e from the other two strains. (PDF)

S2 Fig Response of CD4+, CD8+, and CD4+/CD8+ cells in the spleen to HSV-2 peptides. (PDF)

S3 Fig Response of CD4+, CD8+, and CD4+/CD8+ cells in the lymph nodes to HSV-2 peptides. (PDF)

S1 Data Data for Fig 2. (PDF)

S2 Data Data for Fig 3. (PDF)

S3 Data Datafor Fig 4. (PDF)

S4 Data Data for Fig 5. (PDF)

S5 Data Data for Fig 6. (PDF)

S6 Data Data for Fig 7. (PDF)

S7 Data Data for S2 Fig. (PDF)

S8 Data Data for S3 Fig. (PDF)

S9 Data Data for Fig 8. (PDF)
