# Supplementary material for: A nonhuman primate model for genital herpes simplex virus 2 infection that results in vaginal vesicular lesions, virus shedding, and seroconversion
Source: PLoS Pathog. 2024 Sep 3;20(9):e1012477. doi: 10.1371/journal.ppat.1012477 (PMC11371218; doi:10.1371/journal.ppat.1012477)
Supplement: S1 Fig — (PDF) [file ppat.1012477.s001.pdf]

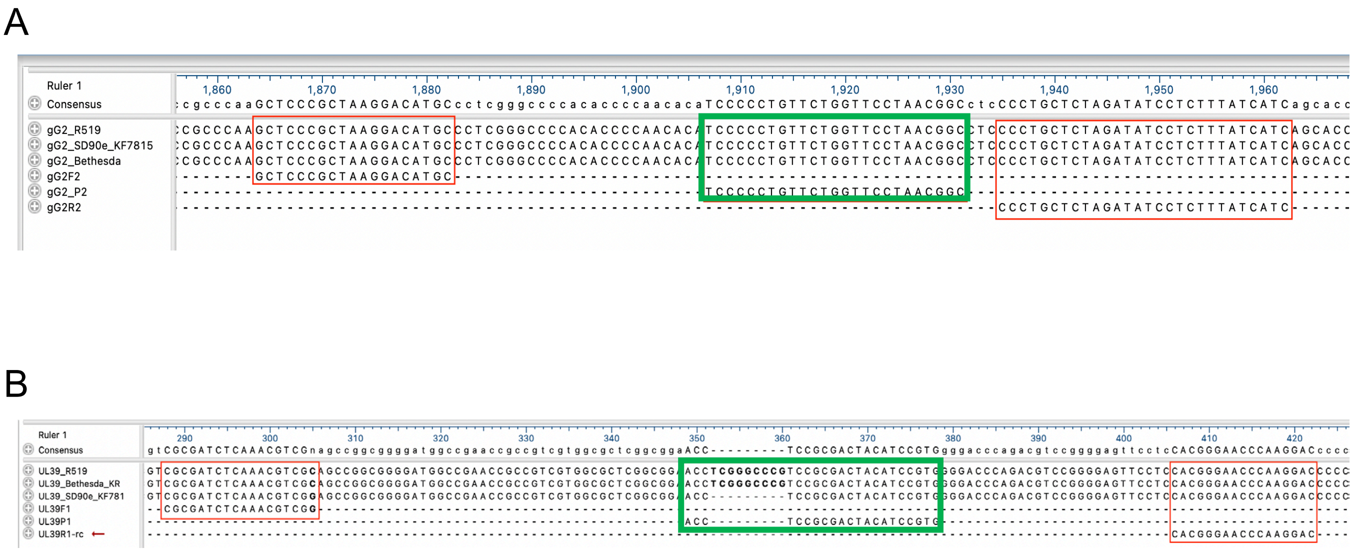

S1 Fig. PCR primers and probes amplify all three HSV-2 strains and distinguish SD90e from the other two strains. Alignment of gG2 primers (red boxes) and probe (green boxes) with gG2 sequences of HSV-2 333 (R519), Bethesda, SD90e (A) and alignment of UL39 primers and probe with UL39 sequences of HSV-2 333 (R519), Bethesda, SD90e (B).
