# Supplementary material for: A nonhuman primate model for genital herpes simplex virus 2 infection that results in vaginal vesicular lesions, virus shedding, and seroconversion
Source: PLoS Pathog. 2024 Sep 3;20(9):e1012477. doi: 10.1371/journal.ppat.1012477 (PMC11371218; doi:10.1371/journal.ppat.1012477)
Supplement: S2 Fig — (PDF) [file ppat.1012477.s002.pdf]

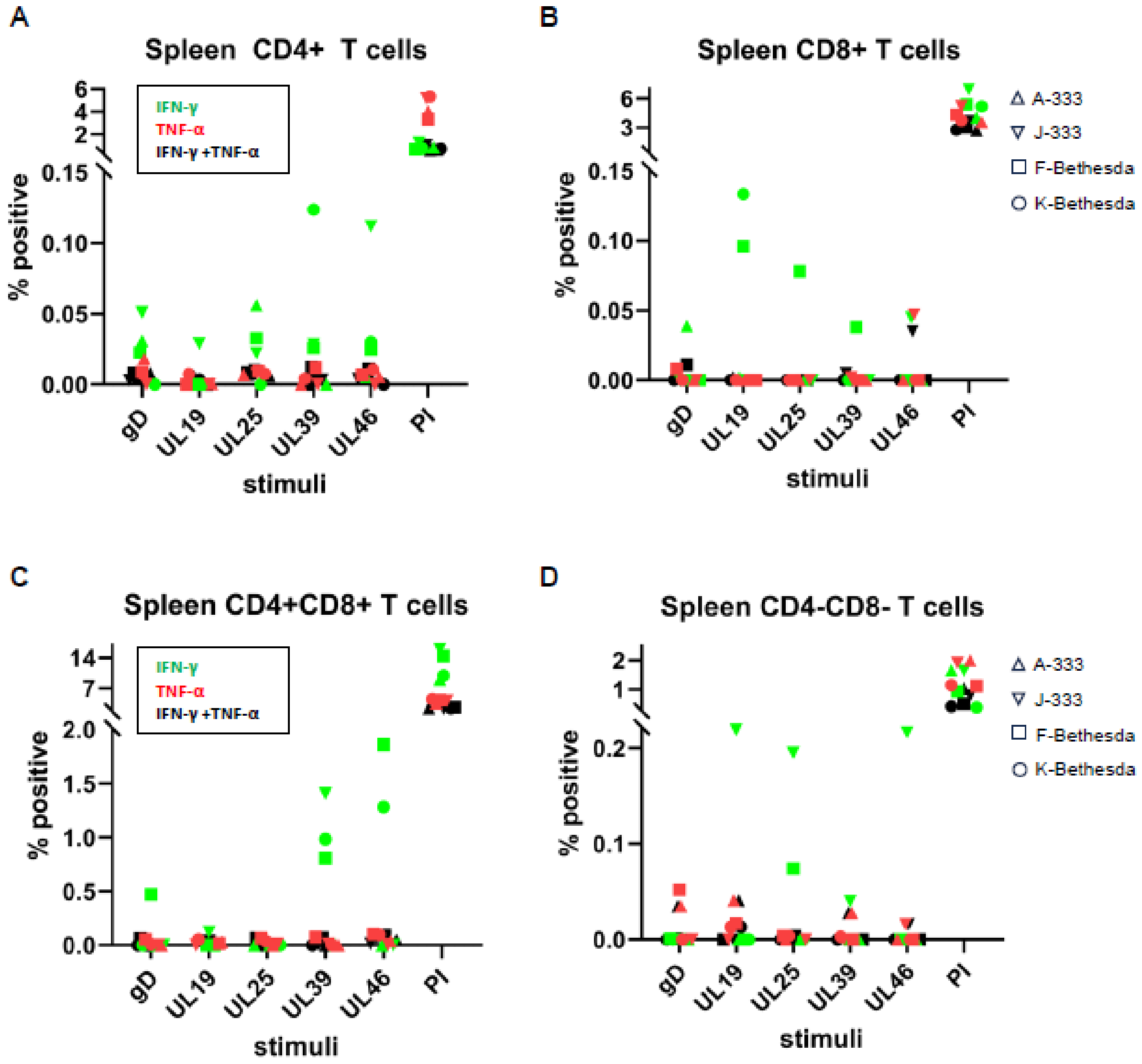

S2 Fig. **Response of CD4+, CD8+, and CD4+/CD8+ cells in the spleen to HSV-2 peptides.** CD4+CD8- (A), CD4-CD8+ (B), CD4+CD8+ [double positive] T cells (C), and CD4-CD8- [double negative] T cells (D) from spleen stained as described in Fig. 7. In the spleen, most of the CD4 and CD8 cells that responded to HSV-2 peptides expressed IFN-γ rather than TNF-α. Data derived from **S7 Data for S2 Fig.pdf**.
