# Supplementary material for: A nonhuman primate model for genital herpes simplex virus 2 infection that results in vaginal vesicular lesions, virus shedding, and seroconversion
Source: PLoS Pathog. 2024 Sep 3;20(9):e1012477. doi: 10.1371/journal.ppat.1012477 (PMC11371218; doi:10.1371/journal.ppat.1012477)
Supplement: S3 Fig — (PDF) [file ppat.1012477.s003.pdf]

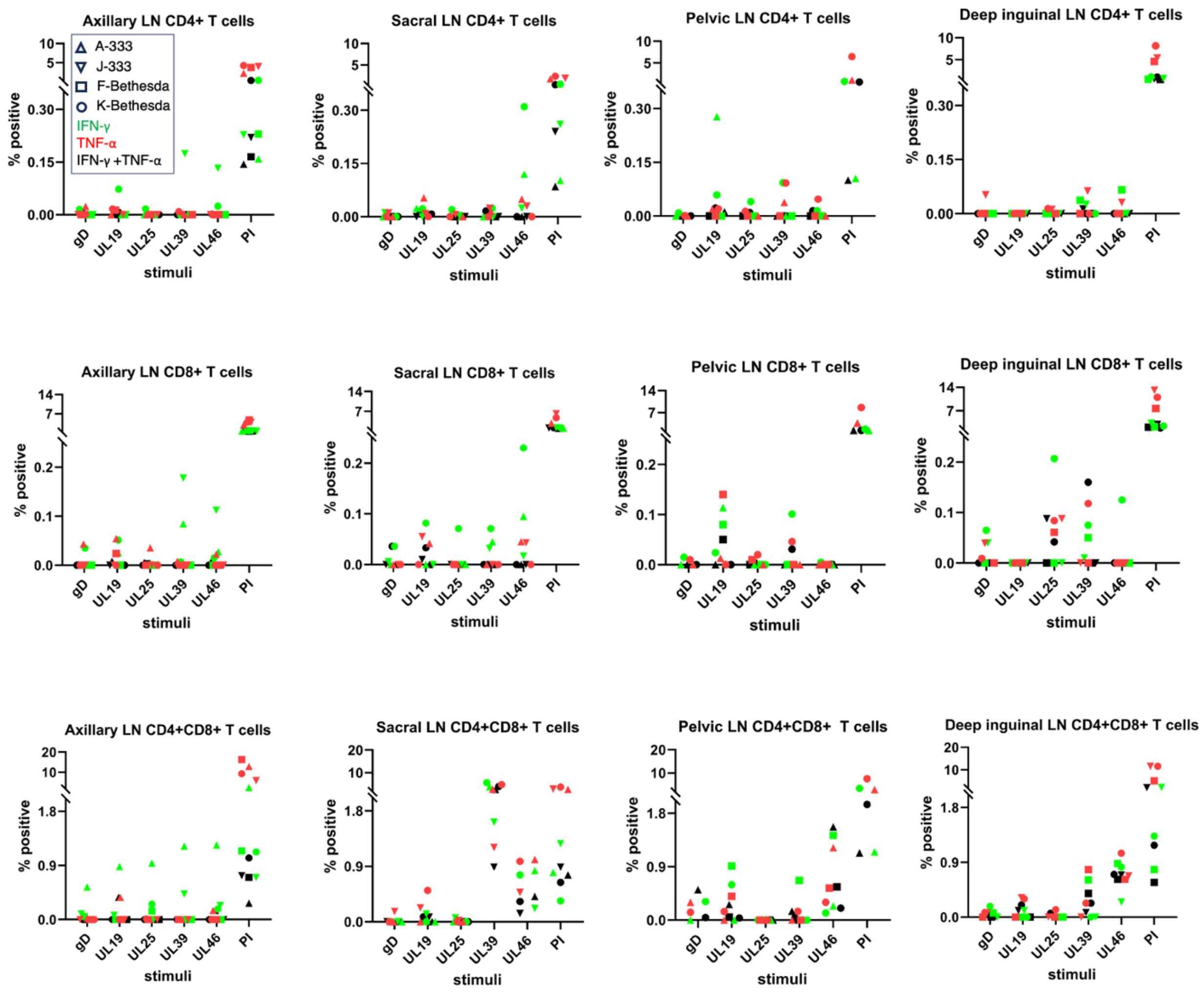

S3 Fig. Response of CD4+, CD8+, and CD4+/CD8+ cells in the lymph nodes to HSV-2 peptides. CD4 (top row), CD8 (middle row), and CD4+ and CD8+ [double positive] T cells (bottom row) cells from lymph nodes of C. apella monkeys stained as described in Fig. 7. Most of T cells from axillary lymph nodes responded to HSV-2 peptide stimulation expressed IFN-γ, less cells expressed TNF-α compared to the cells from other lymph nodes. Data derived from **S8 Data for S3 Fig.pdf**.
