# Supplementary material for: A nonhuman primate model for genital herpes simplex virus 2 infection that results in vaginal vesicular lesions, virus shedding, and seroconversion
Source: PLoS Pathog. 2024 Sep 3;20(9):e1012477. doi: 10.1371/journal.ppat.1012477 (PMC11371218; doi:10.1371/journal.ppat.1012477)
Supplement: S1 Data — (PDF) [file ppat.1012477.s004.pdf]

Data used to generate Fig. 2 Lesion scores over time in C. apella monkeys after primary infection.

Day post-infection	A-333	J-333	F-Bethesda	K-Bethesda
0	0	0	0	0
1	0	0	0	0
2	0	0	0	0
3	0	0	0	0
4	2	0	0	0
7	6	1	0	0
9	4	4	0	0
11	3	6	0	0
14	1	3	0	0
17	0.5	0.5	0	0
21	0	0	0	0
24	0	0	0	0
28	0	0	0	0
31	0	0	0	0
35	1	0	0	0
38	0	0	0	0
43	0	0	0	0
46	0	0	0	0
50	0	0	0	0
53	0	0	0	0
57	0	0	0	0
60	0	0	0	0
64	0	0	0	0
67	0	0	0	0
71	0	0	0	0
74	0	0	0	0
85	0	0	0	0
88	0	0	0	0
92	0	0	0	0
95	0	0	0	0
99	0	0	0	0
102	0	2	0	0
106	0	0	0	0
109	0	0	0	0
