# Supplementary material for: A nonhuman primate model for genital herpes simplex virus 2 infection that results in vaginal vesicular lesions, virus shedding, and seroconversion
Source: PLoS Pathog. 2024 Sep 3;20(9):e1012477. doi: 10.1371/journal.ppat.1012477 (PMC11371218; doi:10.1371/journal.ppat.1012477)
Supplement: S2 Data — (PDF) [file ppat.1012477.s005.pdf]

Data used to generate Fig. 3A C. apella monkeys shed infectious HSV-2 after intravaginal infection

the latent phase of infection

pfu/swab

for Log10 conversion, 0 was replaced with 1

Animal code	A-333	J-333	F-Bethesda	K-Bethesda
day of swab				
0	1	1	1	1
1	5,068,000	672,000	728,000	406,000
2	168,000	95,200	46,200	40,320
3	10,360	15,960	1,624	10,360
4	15,680	18,480	33,880	11,760
7	48,440	52,080	263	30,520
9	22,120	81,200	1	10,080
11	2,520	22,960	1	1,820
14	6	1	Missing	1
28	1	1	1	1
31	1	1	1	1
35	1	1	1	1
38	1	1	1	1
43	1	1	1	1
46	1	1	1	1
50	1	1	1	1
53	1	1	1	1
57	1	1	1	1
60	1	1	1	1
64	1	1	1	1
67	1	1	1	1
71	1	1	1	1
74	1	1	1	1
85	1	1	1	1
88	1	1	1	1
92	1	1	1	1
95	1	1	1	1
99	1	1	1	1
102	1	1	1	1
106	1	1	1	1
109	1	1	1	1

Log 10(pfu/swab)

	A-333	J-333	F-Bethesda	K-Bethesda
0	0	0	0	0
1	7	6	6	6
2	5	5	5	5
3	4	4	3	4
4	4	4	5	4
7	5	5	2	4
9	4	5	0	4
11	3	4	0	3
14	1	0	0	0
28	0	0	0	0
31	0	0	0	0
35	0	0	0	0
38	0	0	0	0
43	0	0	0	0
46	0	0	0	0
50	0	0	0	0
53	0	0	0	0
57	0	0	0	0
60	0	0	0	0
64	0	0	0	0
67	0	0	0	0
71	0	0	0	0
74	0	0	0	0
85	0	0	0	0
88	0	0	0	0
92	0	0	0	0
95	0	0	0	0
99	0	0	0	0
102	0	0	0	0
106	0	0	0	0
109	0	0	0	0

Data used to generate Fig. 3B C. apella monkeys shed HSV-2 DNA after intravaginal infection

gG2 copies/swab

"0" was replaced with "1" for log10 transfer

Animal Code	A-333	J-333	F-Bethesda	K-Bethesda
day of swab				
0	1	1	1	1
1	12,358,486	17,594,882	44,250,000	6,012,625
2	80,250,000	39,250,000	48,500,000	22,645,375
3	280,000,000	102,500,000	202,250,000	18,622,225
4	10,905,181	51,750,000	39,500,000	2,648,775
7	15,166,209	60,000,000	628,999	23,053,225
9	5,103,831	13,896,433	4,866	6,117,125
11	1,266,353	3,004,622	291	4,434,175
14	137,611	37,823	1	6,075
17	131	441	339	1,250
21	1,255	645	1	1
24	530	1	1	1
28	1	1	1	1
31	1	342	1	1
35	1	1	1	1
38	1	1	1	1
43	1	1	1	1
46	1	1	1	1
50	1	1	1	1
53	1	1	1	1
57	1	1	1	1
60	1	1	1	1
64	1	1	1	1
67	1	1	1	1
71	1	1	1	1
74	1	1	1	1
85	1	1	1	1
88	1	1	1	1
92	1	1	1	1
95	1	1	1	1
99	1	1	1	1
102	1	1	1	48
106	1	1	1	1
109	1	1	1	1

Log_{10}(gG2 copies/swab)

	A-333	J-333	F-Bethesda	K-Bethesda
day of swab				
0	0	0	0	0
1	7	7	8	7
2	8	8	8	7
3	8	8	8	7
4	7	8	8	6
7	7	8	6	7
9	7	7	4	7
11	6	6	2	7
14	5	5	0	4
17	2	3	3	3
21	3	3	0	0
24	3	0	0	0
28	0	0	0	0
31	0	3	0	0
35	0	0	0	0
38	0	0	0	0
43	0	0	0	0
46	0	0	0	0
50	0	0	0	0
53	0	0	0	0
57	0	0	0	0
60	0	0	0	0
64	0	0	0	0
67	0	0	0	0
71	0	0	0	0
74	0	0	0	0
85	0	0	0	0
88	0	0	0	0
92	0	0	0	0
95	0	0	0	0
99	0	0	0	0
102	0	0	0	2
106	0	0	0	0
109	0	0	0	0
