# Supplementary material for: A nonhuman primate model for genital herpes simplex virus 2 infection that results in vaginal vesicular lesions, virus shedding, and seroconversion
Source: PLoS Pathog. 2024 Sep 3;20(9):e1012477. doi: 10.1371/journal.ppat.1012477 (PMC11371218; doi:10.1371/journal.ppat.1012477)
Supplement: S3 Data — (PDF) [file ppat.1012477.s006.pdf]

Data used to generate Fig. 4A HSV-2 DNA is detected in the plasma, but not in PBMCs of C. apella monkeys after intravaginal infection

gG2 copies/ml plasma

	0	1	2	3	4	7	9	11	14	Day post-infection
A-333	0.0	235	30100	15163	2174	1012	1282	0.0	121	
J-333	0.0	27	2666	3163	3407	9974	4316	591	351	
F-Bethesda	0.0	2041	9354	7866	1659	824	0.0	0.0	258	
K-Bethesda	0.0	0.0	12797	6078	664	2560	0.0	0.0	0.0	

Data used to generate Fig. 4B HSV-2 DNA is detected in the plasma, but not in PBMCs of C. apella monkeys after intravaginal infection

Monkey PBMC viral DNA load: gG2 copies/10^6 cells

10 ul of PBMC DNA (200 ng) equivorent to 30,732 cells

gG2 copies/10^6 cells=1000000*(gG2 copies/10 ul DNA)/30732

Animal code	0	1	2	3	4	Day post-infection
A-333	0.0	0.0	0.0	0.0	0.0	
J-333	0.0	0.0	0.0	ND	0.0	
F-Bethesda	0.0	0.0	0.0	0.0	0.0	
K-Bethesda	ND	0.0	0.0	0.0	0.0	

ND: PCR not done due to no sample
