# Supplementary material for: A nonhuman primate model for genital herpes simplex virus 2 infection that results in vaginal vesicular lesions, virus shedding, and seroconversion
Source: PLoS Pathog. 2024 Sep 3;20(9):e1012477. doi: 10.1371/journal.ppat.1012477 (PMC11371218; doi:10.1371/journal.ppat.1012477)
Supplement: S4 Data — (PDF) [file ppat.1012477.s007.pdf]

Data used to generate Fig. 5A, vaginal swab virus titer post-reinfection 1

pfu/swab

"0" was replaced with "1" for log10 convertion

Animal code	-5	0	2	3	4	7	9	11	28	day post-reinfection 1
A-333	1	1	1	1	1	1	1	1	1	
J-333	1	1	1	1	1	1	1	1	1	
F-Bethesda	1	1	1	1	1	1	1	1	1	
K-Bethesda	1	1	1	1	1	1	1	1	1	

Log10(pfu/ml)

Animal code	-5	0	2	3	4	7	9	11	28	day post-reinfection 1
A-333	0	0	0	0	0	0	0	0	0	
J-333	0	0	0	0	0	0	0	0	0	
F-Bethesda	0	0	0	0	0	0	0	0	0	
K-Bethesda	0	0	0	0	0	0	0	0	0	

Data used to generate Fig. 5B, vaginal swab virus titer post-reinfection 2

pfu/swab

"0" was replaced with "1" for log10 convertion

Animal code	-5	0	2	3	4	7	9	11	24	day post-reinfection 2
A-333	1	1	3.2	1	1	1	1	1	1	
J-333	1	1	1	1	1	1	1	1	1	
F-Bethesda	1	1	68.8	1	1	1	1	1	1	
K-Bethesda	1	1	640	1	1	1	1	1	1	

Log$_{10}$(pfu/swab)

Animal code	-5	0	2	3	4	7	9	11	24	day post-reinfection 2
A-333	0	0	0.505	0	0	0	0	0	0	
J-333	0	0	0	0	0	0	0	0	0	
F-Bethesda	0	0	1.838	0	0	0	0	0	0	
K-Bethesda	0	0	2.806	0	0	0	0	0	0	

Data used to generate Fig. 5C post-reinfection 3 vaginal swab virus titer

pfu/swab

"0" was replaced with "1" for log10 convertion

Animal code	0	1	2	3	6	8	10	13	15	day post-reinfection 3
A-333	1	1	1	1	1	1	1	1	1	
J-333	1	5	1	1	1	1	1	1	1	
F-Bethesda	1	10	86	1	1	1	1	1	1	
K-Bethesda	1	218	21,920	5,120	1	1	1	1	1	

Log10(pfu/wab)

Animal code	0	1	2	3	6	8	10	13	15	day post-reinfection 3
A-333	0	0	0	0	0	0	0	0	0	
J-333	0	0.68	0	0	0	0	0	0	0	
F-Bethesda	0	0.98	1.94	0	0	0	0	0	0	
K-Bethesda	0	2.34	4.34	3.71	0	0	0	0	0	

Data used to generate Fig. 5D, post-reinfection 4 vaginal swab virus titer

pfu/swab

"0" was replaced with "1" for log10 convertion

Animal code	1	2	3	Day post-reinfection 4
A-333	1,808	2,368	1	
J-333	1	1	1	
F-Bethesda	560	11,200	18,720	
K-Bethesda	328	230	2,352	

Log$_{10}$(pfu/swab)

Animal code	1	2	3	Day post-reinfection 4
A-333	3.26	3.37	0	
J-333	0	0	0	
F-Bethesda	2.75	4.05	4.27	
K-Bethesda	2.52	2.36	3.37	

Data used to generate Fig. 5E, The ratio of the peak vaginal swab HSV-2 titer during primary infection to the highest peak swab titer from all the reinfections is shown

Ratio of primary infection peak titer per swab /re-infection peak titer per swab

Infection	Day p.i.	A-333	J-333	F-Bethesda	K-Bethesda
Primary	1	5,068,000	672,000	728,000	406,000
re-infection 2	2	3.2	0	68.8	640
re-infection 3	2	0	4.8	86.4	21920
re-infection 4	2 or 3	2368	0	18720	2352
**imary/re-inf.**		**2,140**	**140,000**	**38.9**	**18.5**

Log10(Primary titer/reinfection titer)

animal code	A-333	J-333	F-Bethesda	K-Bethesda
log 10 (Primary titer/re-infection titer)	3.3	5.1	1.6	1.3
