# Supplementary material for: A nonhuman primate model for genital herpes simplex virus 2 infection that results in vaginal vesicular lesions, virus shedding, and seroconversion
Source: PLoS Pathog. 2024 Sep 3;20(9):e1012477. doi: 10.1371/journal.ppat.1012477 (PMC11371218; doi:10.1371/journal.ppat.1012477)
Supplement: S5 Data — (PDF) [file ppat.1012477.s008.pdf]

Data used to generate Fig. 6A, Serum HSV-2 neutralizing titers of C. apella monkeys after primary infection and reinfection with HSV-2.

IC_{50}

Animal code	-5	14	28	57	
					day Post-primary infection.
A-333	0	51.1	58.6	49.9	
J-333	0	0.0	19.5	25.3	
F-Bethesda	0	30.4	55.3	87.0	
K-Bethesda	0	24.6	43.7	35.8	

Data used to generate Fig. 6B, Serum HSV-2 neutralizing titers of C. apella monkeys after reinfections 1, 2, and 3

IC_{50}

animal code	Reinfection 1		Reinfection 2		Reinfection 3		
	-5	28	-5	24	-5	28	day post-reinfection
A-333	44.8	29.0	38.3	49.1	29.0	32.3	
J-333	9.7	10.3	5.1	7.2	9.8	10.2	
F-Bethesda	39.3	35.1	43.0	33.2	23.4	30.9	
K-Bethesda	11.0	13.0	11.9	17.5	11.3	24.9	
