# Supplementary material for: A nonhuman primate model for genital herpes simplex virus 2 infection that results in vaginal vesicular lesions, virus shedding, and seroconversion
Source: PLoS Pathog. 2024 Sep 3;20(9):e1012477. doi: 10.1371/journal.ppat.1012477 (PMC11371218; doi:10.1371/journal.ppat.1012477)
Supplement: S6 Data — (PDF) [file ppat.1012477.s009.pdf]

Data used to generate Fig. 7B, PBMC CD4 pre-infection

		gD	UL19	UL25	UL39	UL46	PI
N/A	IFN+TNF+	0.00214	0.00199	0.00422	0.0063	0	2.77
N/A	IFN+TNF+	0	0	0.00296	0.00291	0	3.57
N/A	IFN+TNF+		0.00192	0	0	0.0085	
N/A	total IFN only	0.00492	0	0.00022	0.00908	0	3.23178
N/A	total IFN only	0.00494	0.00591	0.01218	0.00013	0	3.88722
N/A	total IFN only		0.0027	0	0.00138	0.01461	
N/A	total TNF only	0.01314	0	0.00222	0.0103	0	18.447
N/A	total TNF only	0	0	0.00196	0	0	28.628
N/A	total TNF only		0	0	0	0	

Data used to generate Fig. 7C, PBMC CD4 post-infection

		gD	UL19	UL25	UL39	UL46	PI
333	IFN+TNF+	0.034	0.033	0	0.033	0	6.045
Bethesda	IFN+TNF+	0.024	0.00598	0.013	0.11	0.02	3.76
333	IFN+TNF+	0.0146	0	0.0156	0.0126	0.0626	5.0416
Bethesda	IFN+TNF+	0	0.009	0.0073	0	0.01	10
333	total IFN only	0.021	0.064	0	0.02	0	6.182
Bethesda	total IFN only	0.00693	0	0.017	0.134	0.049	4.017
333	total IFN only	0.0306	0	0	0	0.0106	5.3356
Bethesda	total IFN only	0	0	0	0	0	10.404
333	total TNF only	0.05	0.064	0.006	0.035	0	35.032
Bethesda	total TNF only	0.036	0	0.015	0.119	0.022	22.042
333	total TNF only	0.0036	0	0.0536	0.0516	0.2086	26.5076
Bethesda	total TNF only	0	0.045	0.0223	0.022	0.031	36.1

Data used to generate Fig. 7D, PBMC CD8 pre-infection

		gD	UL19	UL25	UL39	UL46	PI
N/A	IFN+TNF+	0.00131	0	0	0	0	2.51
N/A	IFN+TNF+	0.00635	0.00537	0.00897	0	0.00343	2.83
N/A	IFN+TNF+		0	0	0.00051	0.00062	
N/A	total IFN only	0	0	0	0.001	0	9.546
N/A	total IFN only	0.01335	0.00337	0.00497	0	0	6.848
N/A	total IFN only		0	0.01062	0.08451	0.01262	
N/A	total TNF only	0.00131	0.00123	0	0	0	3.73
N/A	total TNF only	0.00612	0.00555	0.01095	0.00168	0.00346	4.6366
N/A	total TNF only		0.00027	0	0	0	

Data used to generate Fig. 7E, PBMC CD8 post-infection

		gD	UL19	UL25	UL39	UL46	PI
333	IFN+TNF+	0	0	0	0	0	4.63
Bethesda	IFN+TNF+	0.00491	0	0	0	0	11.4
333	IFN+TNF+	0.014	0	0	0.049	0.043	5.67
Bethesda	IFN+TNF+	0.015	0.00132	0.0001	0.02	0.04	8.155
333	total IFN only	0.011	0	0	0	0.00897	7.44
Bethesda	total IFN only	0.00473	0	0	0.00002	0.00491	18.75491
333	total IFN only	0.069	0	0	0.049	0.057	10.36
Bethesda	total IFN only	0.02158	0.01432	0.0201	0.04	0.098	17.225
333	total TNF only	0	0	0	0	0.00897	9.44
Bethesda	total TNF only	0.00473	0.00485	0.00491	0.00002	0.00015	18.44491
333	total TNF only	0.003	0.004	0	0.062	0.046	13.159
Bethesda	total TNF only	0.023	0.01532	0.0051	0.01992	0.067	14.59

Data used to generate Fig. 7F, dessociated lymph node cells CD4+

	axillary	pelvic	sacral	inguinal	spleen	PBMC	naive PBMC
333	0.02386	0.335	0.2609		0.12563	0.26	0.03988
333	0.30657		0.11883	0.184	0.25011	0.3586	0.01869
Bethesda	0.013	0		0.103	0.14341	0.39893	0.02512
Bethesda	0.15466	0.38867	0.37643	0.014	0.19103	0.1203	

Data used to generate Fig. 7G, dessociated lymph node cells CD8+

	axillary	pelvic	sacral	inguinal	spleen	PBMC	naive PBMC
333	0.30232	0.127	0.227		0.041	0.02894	0.00354
333	0.29047		0.153	0.177	0.094	0.29	0.10802
Bethesda	0.024	0.23		0.111	0.22	0.02432	0.04945
Bethesda	0.10982	0.221	0.49	0.683	0.13331	0.32434	
