# Supplementary material for: A nonhuman primate model for genital herpes simplex virus 2 infection that results in vaginal vesicular lesions, virus shedding, and seroconversion
Source: PLoS Pathog. 2024 Sep 3;20(9):e1012477. doi: 10.1371/journal.ppat.1012477 (PMC11371218; doi:10.1371/journal.ppat.1012477)
Supplement: S7 Data — (PDF) [file ppat.1012477.s010.pdf]

Data used to generate S2 Fig. A Spleen CD4+ T cells

		gD	UL19	UL25	UL39	UL46	PI
333	IFN+TNF+	0.00778	0.00597	0.00614	0	0.00541	0.66
Bethesda	IFN+TNF+	0.00837	0	0.00869	0.012	0.011	0.61
333	IFN+TNF+	0.00307	0	0.01	0.00336	0.00389	1.04
Bethesda	IFN+TNF+	0.00378	0.00357	0.00726	0.00386	0	0.7
333	total IFN only	0.03078	0.00197	0.05614	0	0.00541	0.84
Bethesda	total IFN only	0.02237	0	0.03269	0.026	0.025	0.674
333	total IFN only	0.05107	0.029	0.022	0.02836	0.11189	1.238
Bethesda	total IFN only	0	0	0	0.12386	0.03	0.74
333	total TNF only	0.0184	0.00059	0.0069	0	0.00544	4.05462
Bethesda	total TNF only	0.00896	0	0.0096	0.01193	0.00686	3.34222
333	total TNF only	0	0	0.00779	0	0	5.18109
Bethesda	total TNF only	0.00785	0.00721	0.00739	0.00422	0.0105	5.3365

Data used to generate S2 Fig. B Spleen CD8+ T cells

		gD	UL19	UL25	UL39	UL46	PI
333	IFN+TNF+	0	0.002	0	0	0	2.715
Bethesda	IFN+TNF+	0.011	0	0	0	0	3.044
333	IFN+TNF+	0	0	0	0.005	0.035	3.654
Bethesda	IFN+TNF+	0	0	0	0	0	2.757
333	total IFN only	0.039	0.002	0	0	0	4.015
Bethesda	total IFN only	0	0.096	0.078	0.038	0	5.404
333	total IFN only	0	0	0	0	0.045	6.934
Bethesda	total IFN only	0	0.13331	0	0	0	5.187
333	total TNF only	0	0	0	0	0	3.55993
Bethesda	total TNF only	0.008	0	0	0	0	4.321
333	total TNF only	0	0	0	0.002	0.047	5.199
Bethesda	total TNF only	0	0	0	0	0	3.74711

Data used to generate S2 Fig. C Spleen CD4+CD8+ T cells

		gD	UL19	UL25	UL39	UL46	PI
333	IFN+TNF+	0	0.042	0	0	0.049	2.396
Bethesda	IFN+TNF+	0.063	0.041	0.064	0.067	0.091	2.61
333	IFN+TNF+	0	0	0	0.01	0.013	2.351
Bethesda	IFN+TNF+	0.001	0.038	0.022	0	0.081	2.411
333	total IFN only	0	0	0	0	0	9.016
Bethesda	total IFN only	0.473	0	0	0.807	1.861	14.48
333	total IFN only	0	0.122	0	1.41	0	16.021
Bethesda	total IFN only	0	0	0	0.983	1.281	9.911
333	total TNF only	0	0.03	0	0	0.037	3.484
Bethesda	total TNF only	0.052	0.017	0.066	0.076	0.103	4.296
333	total TNF only	0	0	0.01178	0.01059	0.00578	4.17378
Bethesda	total TNF only	0.0089	0.053	0.037	0	0.098	4.501

Data used to generate S2 Fig. D Spleen CD4-CD8- T cells

		gD	UL19	UL25	UL39	UL46	PI
333	IFN+TNF+	0.035	0.041	0	0.028	0	1.02
Bethesda	IFN+TNF+	0.001	0	0.004	0	0	0.495
333	IFN+TNF+	0	0	0	0	0.016	0.78
Bethesda	IFN+TNF+	0	0.013	0	0	0	0.4
333	total IFN only	0	0	0	0	0	1.66
Bethesda	total IFN only	0.001	0	0.074	0	0	0.925
333	total IFN only	0	0.219	0.195	0.04	0.216	1.64
Bethesda	total IFN only	0	0	0	0	0	0.37
333	total TNF only	0.035	0.041	0	0.028	0	2
Bethesda	total TNF only	0.052	0.017	0.004	0	0	1.115
333	total TNF only	0	0	0	0	0.016	1.91
Bethesda	total TNF only	0	0.013	0.004	0.003	0	1.14
