# Supplementary material for: A nonhuman primate model for genital herpes simplex virus 2 infection that results in vaginal vesicular lesions, virus shedding, and seroconversion
Source: PLoS Pathog. 2024 Sep 3;20(9):e1012477. doi: 10.1371/journal.ppat.1012477 (PMC11371218; doi:10.1371/journal.ppat.1012477)
Supplement: S8 Data — (PDF) [file ppat.1012477.s011.pdf]

Data used to generate S3 Fig. Axilllry LN CD4+ T cells

		gD	UL19	UL25	UL39	UL46	PI
333	IFN+TNF+	0	0	0	0	0	0.144
Bethesda	IFN+TNF+	0	0.002	0	0	0	0.165
333	IFN+TNF+	0	0	0	0	0	0.22
Bethesda	IFN+TNF+	0.00518	0.00658	0	0	0.00141	0.36667
333	total IFN only	0	0	0	0	0	0.159
Bethesda	total IFN only	0	0	0	0	0	0.23
333	total IFN only	0	0	0	0.174	0.13257	0.228
Bethesda	total IFN only	0.01518	0.07258	0.01638	0	0.02441	0.42067
333	total TNF only	0.02386	0	0	0	0	2.261
Bethesda	total TNF only	0	0.013	0	0	0	3.75
333	total TNF only	0	0	0	0	0	3.99272
Bethesda	total TNF only	0.00086	0.01658	0	0.00867	0	4.26667

Data used to generate S3 Fig. Sacral LN CD4+ T cells

		gD	UL19	UL25	UL39	UL46	PI
333	IFN+TNF+	0	0.018	0	0	0	0.085
333	IFN+TNF+	0	0.00755	0	0.016	0	0.42
Bethesda	IFN+TNF+	0	0	0	0	0	0.24
333	total IFN only	0	0.0239	0	0	0.12	0.102
333	total IFN only	0	0.02255	0.02	0.02388	0.31	0.53
Bethesda	total IFN only	0.01119	0	0.00319	0	0.02419	0.26019
333	total TNF only	0	0.053	0	0.015	0.049	1.765
333	total TNF only	0	0	0	0	0	2.367
Bethesda	total TNF only	0.011	0.0089	0.00636	0.024	0.03	1.92

Data used to generate S3 Fig. Pelvic LN CD4+ T cells

		gD	UL19	UL25	UL39	UL46	PI
Bethesda	IFN+TNF+	0	0.022	0.01	0	0.015	0.6
333	IFN+TNF+	0	0.012	0	0	0	0.1
Bethesda	IFN+TNF+		0	0	0	0	
Bethesda	total IFN only	0.00867	0.059	0.04	0.093	0.015	0.74
333	total IFN only	0	0.277	0	0	0	0.105
Bethesda	total IFN only		0	0	0	0	
Bethesda	total TNF only	0.001	0.02	0.013	0.092	0.047	6.452
333	total TNF only	0	0.02	0	0.038	0	1.055
Bethesda	total TNF only		0	0	0	0	

Data used to generate S3 Fig. Deep inguinal LN CD4+ T cells

		gD	UL19	UL25	UL39	UL46	PI
Bethesda	IFN+TNF+	0	0	0	0	0	0.45
333	IFN+TNF+	0	0	0	0.013	0	0.61
Bethesda	IFN+TNF+	0	0	0	0	0	0.97
Bethesda	total IFN only	0	0	0	0.037	0.066	0.475
333	total IFN only	0	0	0	0.026	0	0.654
Bethesda	total IFN only	0	0	0	0	0	1.006
Bethesda	total TNF only	0	0	0	0	0	4.589
333	total TNF only	0.052	0	0.012	0.063	0.031	5.343
Bethesda	total TNF only	0	0	0.014	0	0	8.161

Data used to generate S3 Fig. Axilllry LN CD8+ T cells

		gD	UL19	UL25	UL39	UL46	PI
333	IFN+TNF+	0.00154	0.00854	0.00554	0	0.00654	0.56054
Bethesda	IFN+TNF+	0	0	0	0	0	0.75
333	IFN+TNF+	0	0	0.003	0	0	0.583
Bethesda	IFN+TNF+	0	0	0	0	0	0.684
333	total IFN only	0	0.02554	0	0.08454	0.02754	0.66454
Bethesda	total IFN only	0	0	0	0	0	0.881
333	total IFN only	0	0	0	0.178	0.11247	0.588
Bethesda	total IFN only	0.035	0.05152	0.001	0.007	0.0153	0.826
333	total TNF only	0.04354	0.05454	0.03554	0.00854	0.02254	2.91054
Bethesda	total TNF only	0	0.024	0	0	0	4.72
333	total TNF only	0	0	0	0	0	3.817
Bethesda	total TNF only	0	0	0	0	0	3.876

Data used to generate S3 Fig. Sacral LN CD8+ T cells

		gD	UL19	UL25	UL39	UL46	PI
333	IFN+TNF+	0	0	0	0	0	0.96
333	IFN+TNF+	0.036	0.033	0	0	0	1.15
Bethesda	IFN+TNF+	0	0.01	0	0	0	1.064
333	total IFN only	0	0	0	0.045	0.095	1.025
333	total IFN only	0.036	0.082	0.071	0.071	0.23	1.197
Bethesda	total IFN only	0.006	0	0	0.032	0.017	1.182
333	total TNF only	0	0.042	0	0	0.045	2.81
333	total TNF only	0	0	0	0	0	4.72
Bethesda	total TNF only	0	0.055	0	0	0.043	6.128

Data used to generate S3 Fig. Pelvic LN CD8+ T cells

		gD	UL19	UL25	UL39	UL46	PI
Bethesda	IFN+TNF+	0	0	0	0.031	0	1.381
333	IFN+TNF+	0	0	0.004	0	0	1.352
Bethesda	IFN+TNF+		0.05	0	0	0	
Bethesda	total IFN only	0.015	0.024	0	0.101	0.005	1.751
333	total IFN only	0	0.114	0	0	0	1.342
Bethesda	total IFN only		0.08	0	0	0	
Bethesda	total TNF only	0.01	0	0.02	0.046	0	8.616
333	total TNF only	0	0.013	0	0	0	3.716
Bethesda	total TNF only		0.14	0.01	0	0	

Data used to generate S3 Fig. Deep inguinal LN CD8+ T cells

		gD	UL19	UL25	UL39	UL46	PI
Bethesda	IFN+TNF+	0	0	0	0	0	2.09
333	IFN+TNF+	0	0	0.088	0	0	2.93
Bethesda	IFN+TNF+	0	0	0.042	0.16	0	1.87
Bethesda	total IFN only	0	0	0	0.05	0	2.2
333	total IFN only	0.04	0	0	0.01	0	3.23
Bethesda	total IFN only	0.065	0	0.207	0.075	0.125	2.405
Bethesda	total TNF only	0	0	0.061	0	0	7.681
333	total TNF only	0.039	0	0.088	0	0	13.13
Bethesda	total TNF only	0.009	0	0.084	0.118	0	11.018

Data used to generate S3 Fig. Axilllry LN CD4+CD8+ T cells

		gD	UL19	UL25	UL39	UL46	PI
333	IFN+TNF+	0	0.37	0	0	0.15	0.27
Bethesda	IFN+TNF+	0	0	0	0	0	0.7
333	IFN+TNF+	0	0	0	0	0	0.73
Bethesda	IFN+TNF+	0.004	0	0	0	0	1.024
333	total IFN only	0.54	0.88	0.938	1.22	1.24	2.35
Bethesda	total IFN only	0.05	0	0.14	0	0	1.14
333	total IFN only	0.095	0.07	0	0.425	0.234	0.7
Bethesda	total IFN only	0	0.004	0.256	0	0.175	1.124
333	total TNF only	0.02	0.37	0	0	0.15	12.87
Bethesda	total TNF only	0	0	0	0	0	16.29
333	total TNF only	0	0	0	0	0	5.79
Bethesda	total TNF only	0	0	0.011	0	0	9.239

Data used to generate S3 Fig. Sacral LN CD4+CD8+ T cells

		gD	UL19	UL25	UL39	UL46	PI
333	IFN+TNF+	0	0	0	2.65	0.41	0.76
333	IFN+TNF+	0	0.08	0	3.9	0.33	0.64
Bethesda	IFN+TNF+	0	0.077	0	0.887	0.137	0.887
333	total IFN only	0	0	0	3.77	0.83	0.8
333	total IFN only	0	0	0	5.64	0.76	0.34
Bethesda	total IFN only	0	0.127	0.065	1.617	0.217	1.267
333	total TNF only	0	0	0	2.76	1.01	2.62
333	total TNF only	0	0.51	0	4.75	0.98	3.68
Bethesda	total TNF only	0.167	0.227	0.023	1.207	0.477	2.807

Data used to generate S3 Fig. Pelvic LN CD4+CD8+ T cells

		gD	UL19	UL25	UL39	UL46	PI
Bethesda	IFN+TNF+	0.044	0.035	0	0.037	0.2	1.95
333	IFN+TNF+	0.51	0.26	0	0.15	1.57	1.13
Bethesda	IFN+TNF+		0.05	0	0	0.56	
Bethesda	total IFN only	0.314	0.595	0	0	0.12	3.39
333	total IFN only	0	0	0	0	0.24	1.15
Bethesda	total IFN only		0.91	0	0.67	1.43	
Bethesda	total TNF only	0.132	0.145	0	0.147	0.299	7.58
333	total TNF only	0.3	0	0	0	1.22	2.85
Bethesda	total TNF only		0.4	0	0	0.54	

Data used to generate S3 Fig. Deep inguinal LN CD4+CD8+ T cells

		gD	UL19	UL25	UL39	UL46	PI
Bethesda	IFN+TNF+	0	0	0	0.39	0.62	0.57
333	IFN+TNF+	0	0.11	0	0.076	0.69	2.12
Bethesda	IFN+TNF+	0.078	0.2	0.062	0.23	0.7	1.18
Bethesda	total IFN only	0.07	0	0	0.61	0.88	0.78
333	total IFN only	0	0.11	0	0	0.25	2.19
Bethesda	total IFN only	0.178	0	0	0	0.82	1.33
Bethesda	total TNF only	0	0	0	0.78	0.62	5.12
333	total TNF only	0.07	0.32	0	0	0.678	11.57
Bethesda	total TNF only	0.078	0.3	0.124	0.23	1.05	11.58
