# Supplementary material for: A nonhuman primate model for genital herpes simplex virus 2 infection that results in vaginal vesicular lesions, virus shedding, and seroconversion
Source: PLoS Pathog. 2024 Sep 3;20(9):e1012477. doi: 10.1371/journal.ppat.1012477 (PMC11371218; doi:10.1371/journal.ppat.1012477)
Supplement: S9 Data — (PDF) [file ppat.1012477.s012.pdf]

Data used to generate Fig. 8A HSV-2 grows to higher titers in C. apella primary fibroblasts than in rhesus macaque primary fibroblasts

Titer on Vero cell (pfu/ml)

Virus	cell type	Adjusted 0 hr. sample titer pfu/ml	Group 1 (8 hr.) average pfu/ml	Group 3 (26 Hr.) average pfu/ml
		0	8	26
R519	Vero	16,533	94	4,956,667
R519	Hu DLM	16,533	174	2,000,000
R519	Hu NC	16,533	108	1,500,000
R519	Cebus A	16,533	140	352,000
R519	Cebus F	16,533	148	152,667
R519	Rhesus 9	16,533	238	6,360
R519	Rhesus 3	16,533	286	131
Bethesda	Vero	7,840	98	1,493,333
Bethesda	Hu DLM	7,840	118	900,000
Bethesda	Hu NC	7,840	214	525,000
Bethesda	Cebus A	7,840	106	309,000
Bethesda	Cebus F	7,840	148	102,000
Bethesda	Rhesus 9	7,840	164	4,107
Bethesda	Rhesus 3	7,840	130	144
SD90e	Vero	15,200	250	3,350,691
SD90e	Hu DLM	15,200	162	2,020,000
SD90e	Hu NC	15,200	278	1,210,000
SD90e	Cebus A	15,200	238	651,500
SD90e	Cebus F	15,200	300	239,333
SD90e	Rhesus 9	15,200	390	5,680
SD90e	Rhesus 3	15,200	348	241

Log10(pfu/ml)

Virus	cell type	Adjusted 0 hr. sample titer pfu/ml	Group 1 (8 hr.) average pfu/ml	Group 3 (26 Hr.) average pfu/ml
		0	8	26
R519	Vero	4.2184	1.9731	6.6952
R519	Hu DLM	4.2184	2.2405	6.3010
R519	Hu NC	4.2184	2.0334	6.1761
R519	Cebus A	4.2184	2.1461	5.5465
R519	Cebus F	4.2184	2.1703	5.1837
R519	Rhesus 9	4.2184	2.3766	3.8035
R519	Rhesus 3	4.2184	2.4564	2.1162
Bethesda	Vero	3.8943	1.9912	6.1742
Bethesda	Hu DLM	3.8943	2.0719	5.9542
Bethesda	Hu NC	3.8943	2.3304	5.7202
Bethesda	Cebus A	3.8943	2.0253	5.4900
Bethesda	Cebus F	3.8943	2.1703	5.0086
Bethesda	Rhesus 9	3.8943	2.2148	3.6135
Bethesda	Rhesus 3	3.8943	2.1139	2.1584
SD90e	Vero	4.1818	2.3979	6.5251
SD90e	Hu DLM	4.1818	2.2095	6.3054
SD90e	Hu NC	4.1818	2.4440	6.0828
SD90e	Cebus A	4.1818	2.3766	5.8139
SD90e	Cebus F	4.1818	2.4771	5.3790
SD90e	Rhesus 9	4.1818	2.5911	3.7543
SD90e	Rhesus 3	4.1818	2.5416	2.3826
